# MACC1 ablation suppresses the dedifferentiation process of non-CSCs in lung cancer through stabilizing KLF4

**DOI:** 10.1038/s41420-024-02256-0

**Published:** 2024-12-18

**Authors:** Zhuoshi Li, Shiqing Wang, Tao Guo, Xinyi Yan, Chaoqun Chen, Wenjing Zhang, Jinyao Zhao, Jinrui Zhang, Shilei Zhao, Yang Wang, Yangfan Qi, Chundong Gu

**Affiliations:** 1 Department of Thoracic Surgery, The First Affiliated Hospital of Dalian Medical University, Dalian, 116044 China; 2 Lung Cancer Diagnosis and Treatment Center of Dalian, The First Affiliated Hospital of Dalian Medical University, Dalian, Liaoning 116011 China; 3 Department of Nephrology, Dalian Municipal Central Hospital, Dalian University of Technology, Dalian, 116033 China; 4 Institute of Cancer Stem Cell, Dalian Medical University, Dalian, 116044 China

**Keywords:** Oncogenes, Cancer stem cells

## Abstract

Metastasis-associated in colon cancer-1 (MACC1) was identified as a new player in lung cancer development, and some stemness-related genes can be novel transcriptional targets of MACC1. Cancer stem cells (CSCs) are responsible for sustaining tumorigenesis and plasticity. Both CSCs and non-CSCs are plastic and capable of undergoing phenotypic transition, especially the dedifferentiation of non-CSCs switch to CSC-like cells. However, the precise role of MACC1 during this process is largely unknown. Here, we showed that MACC1 promoted the transition from non-CSC to CSC in lung cancer. We found MACC1 was overexpressed in stemness enriched cells, enhancing the transition from no-CSCs to CSCs, while short-hairpin RNA-mediated Knockdown of MACC1 impaired this process. High-throughput sequencing and tumor specimen analysis revealed that MACC1 was negative correlated with Krüppel-like factor 4 (KLF4) expression level, which acts as a negative stemness regulator in lung cancer. Mechanistically, MACC1 delays the degradation of KLF4 mRNA by repressing the expression of microRNA-25, thereby promoting the KLF4 mRNA stabilization at the post-transcriptional level. Collectively, our findings may facilitate efforts to promote the development of precision targeted therapy for cancer stem cells in lung cancer.

## Introduction

Lung cancer is one of the most common malignant tumors in the world, with the highest mortality rate of malignant tumors [1]. Despite considerable progress made in diagnosis and precise treatment, it is undeniable that the 5-year survival rate of lung cancer patients is still between 4% and 17% depending on stage and regional differences [2]. Overcoming drug resistance and recurrence of lung cancer remains a big challenge. Accumulating evidence suggests that cancer stem cells (CSCs) play a pivotal role in creating the problem [3, 4]. According to the CSC model, tumor initiation, growth, and maintenance are all driven by a small population of cells, known as tumor-initiating CSCs [5, 6]. So far, lots of studies were conducted to identify CSCs by biomarkers [7]. For example, lung carcinomas with high ALDH1^+^ activity are capable of self-renewal, generating tumors and multidirectional differentiation [8–10] characterized as CSCs. Nevertheless, little is known about the molecular mechanism determining the cell fate of CSCs. Traditional cancer stem cell models point out that stem cells are at the top of the cell hierarchy and generate a new stem cell and a daughter cell through unequal division [5]. In recent years, it has been hypothesized that well-differentiated non-cancer stem cells can also regain stem-like properties into cancer stem cells in some ways [11, 12], which are considered to be responsible for cancer metastasis and relapse, yet no impactful strategies were developed to restrain the process. Exploring and figuring out the key factor or mechanism of non-CSCs dedifferentiation has become an urgent problem to be solved.

As one of the important genes of induced pluripotent stem cells, KLF4 with Oct3/4, Sox2, c-Myc were demonstrated to drive induction of pluripotent stem cells from mouse embryonic or adult fibroblasts under ES cell culture conditions [13]. However, KLF4 was content dependent stemness factor as a new insight emerging with research progression. For example, KLF4 inhibits the pancreatic cancer cells stem-like properties by suppressing CD44 [14]. Similarly, polarity protein NUMBL expressed in normal neural progenitor cells maintains progenitor-like characteristics in lung cancer by inhibiting KLF4, and restores KLF4 expression level can destroy progenitor cell subsets, thereby inhibiting the growth of lung cancer cells [15]. KLF4 can also inhibit nasopharyngeal carcinoma stem cells, reverse epithelial-mesenchymal transition, and block the progression of nasopharyngeal carcinoma [16]. Although stable KLF4 could suppress lung tumorigenesis [17], the function for lung cancer stem cells is largely unknown.

Metastasis-associated in colon cancer 1 (MACC1) was original identified as a transcription factor of c-MET, which activates the HGF/Met signal pathway and induces colon cancer cell metastasis and invasion [18]. Nowadays, MACC1 has been confirmed to be a key regulator of malignant proliferation and metastasis in various solid tumor [19–23], including lung cancer [24–26]. For example, MACC1 promotes the epithelial-mesenchymal transition (EMT) process by binding to SNAIL [23] that mediates apoptosis through STAT1/3 and Akt/β-catenin signaling pathway [27, 28] and induces tumor growth against metabolic stress by facilitating the Warburg effect [29]. Besides, MACC1 has been reported to promote tumor immune escape via upregulating PDL1 (Programmed cell death ligand 1) expression [30]. In addition, MACC1 can also induce tumor progression in transgenic mice and colorectal cancer patients by activating stemness protein markers Oct4 and Nanog [31]. Knockdown MACC1 was demonstrated to suppress sphere-formation of cervical cancer cells and down regulated OCT4 and Nanog [22]. Above findings suggest MACC1 has a potential oncogenic role in tumor progression. However, the potential mechanism for MACC1-mediated lung cancer malignant proliferation remains uncertain, particularly the role of MACC1 participating in the process of non-CSCs dedifferentiation is still poorly characterized. In this study, we will explore the potential role and mechanism of MACC1 on the dedifferentiation of non-cancer stem cells.

## Result

### MACC1 is upregulated in stemness-enriched lung cancer cells population

**Fig. 1:**
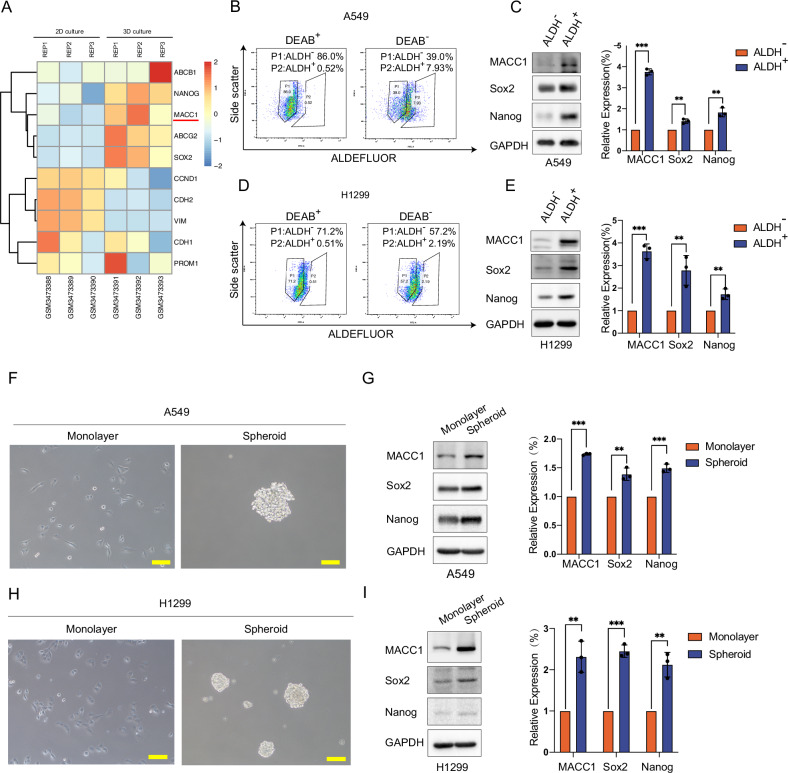
MACC1 is upregulated in stemness-enriched lung cancer cells population. **A** Heatmap displayed MACC1 upregulated in 3D cultured lung cancer cell line TUM622 using genome mRNA expression profiling data (GSE12538 from GEO database). ALDH flow cytometric sorting analysis of ALDH^−^ (P1) and ALDH^+^ (P2) cell subpopulations proportion in A549 (**B**) and H1299 (**D**) cell lines. The protein level of MACC1 and stemness marker Sox2, Nanog were examined in A549 (**C**) or H1299 (**E**) cells after sorting into ALDH^+^ and ALDH^-^ subpopulations, column graph represented relative quantification of protein, three replicates were shown for each group **P < 0.01, ***P < 0.001, two-tailed Student’s t-tests. Error bars represented mean ± SD. Lung cancer cell lines A549 (**F**) and H1299 (**H**) cultured with adherent (monolayer) or suspended (Spheroid) for 7 days. The protein level of MACC1 and stemness marker Sox2, Nanog were examined in A549 (**G**) or H1299 (**I**), column graph represented relative quantification of protein, three replicates were shown for each group. **P < 0.01, ***P < 0.001, two-tailed Student’s t-tests. Error bars: mean ± SD.

Stem cells genes have been shown to be enriched in 3-dimensional (3D) culture (also known as spheroid culture) [32, 33]. To further explore the connection between MACC1 and lung cancer stem cells, we firstly analyzed the gene expression profiles of lung cancer cells TUM622 under 3D and 2D culture conditions. (GSE122538 from GEO database) [34]. As shown in the heap map (Fig. 1A), we found that Sox2, Nanog and other genes mediated stem-like properties increased in the 3D cultured cell subpopulation, especially, MACC1 gene probe signal intensity was significantly higher than 2D culture subpopulation. To further verify the correlation between MACC1 and lung cancer CSCs, we used ALDH flow cytometry sorting technology and sphere formation assay to isolate lung cancer cells with stem-like properties, and identify MACC1 expression levels with rest non-stem cell subsets. A549 and H1299 lung cancer cells were sorted by ALDH flow cytometry (Fig. 1B, D), the ALDH^−^ and ALDH^+^ cell subsets were collected respectively. As expected, Sox2 and Nanog known as the traditional CSCs biomarkers were elevated in the ALDH^+^ subsets, noteworthy, MACC1 protein expression level was higher in ALDH^+^ than ALDH^-^ subpopulations (Fig. 1C, E). In the meanwhile, we conducted sphere formation assay to enrich the spheroid cells in A549 and H1299 lung cancer cells, and the adherent cultured cells were used as a control (Fig. 1F, H). We compared expression differences of traditional cancer stem biomarker Sox2, Nanog and MACC1 between the two cultured conditions. In addition to Sox2 and Nanog, we also found that MACC1 protein levels were also significantly up-regulated in stemness-enriched subpopulations (Fig. 1G, I), which is consistent with gene expression profiles analysis.

### MACC1 is a pivotal regulator for the non-CSCs dedifferentiation of lung cancer in vitro

**Fig. 2:**
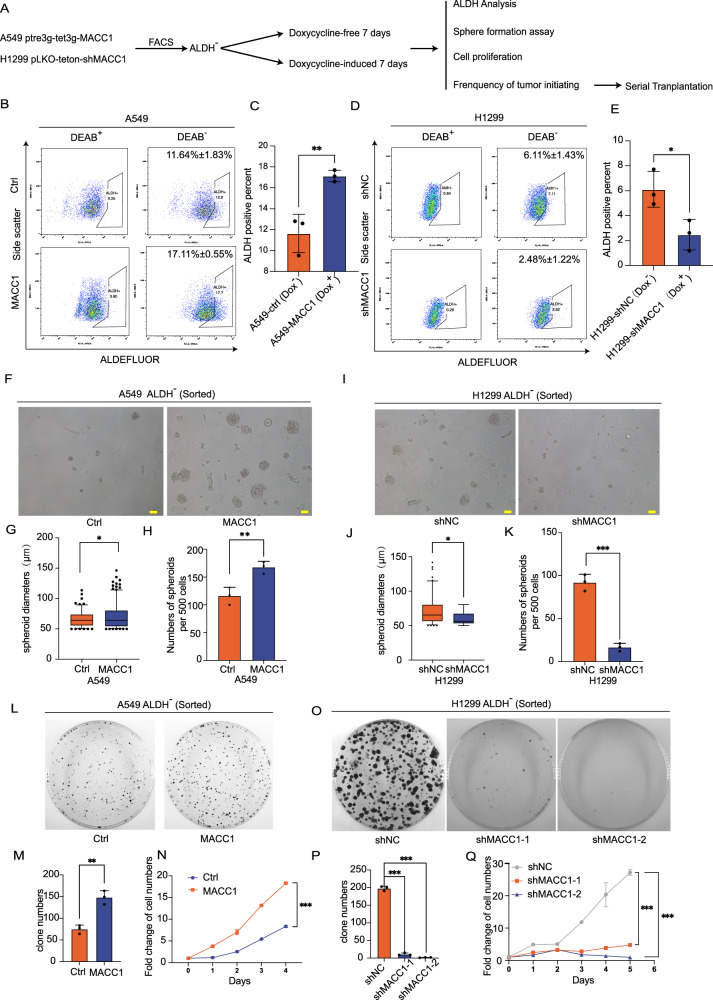
MACC1 is a pivotal regulator for the non-CSCs dedifferentiation of lung cancer in vitro. **A** Schematic outline of the experimental procedure in Dox-inducible MACC1 overexpression cells (A549) or knockdown cells (H1299). A549 ALDH^-^ (sorted) and H1299 ALDH^-^ (sorted) were cultured in the absence or presence of Dox for 7 days, **B**–**E** the percentage of ALDH^+^ cells were determined using FACS. comparison of sphere-forming ability (**F**–**K**), colony-forming numbers and cell viability (**L**–**Q**) were analyzed following MACC1 interference (absence or presence of Dox). *P < 0.05 **P < 0.01***P < 0.001, two-tailed Student’s t-tests and ANOVA. Error bars: mean ± SD. Scale bars, 100 μm.

Given that the CSC subpopulation in cancer can be maintained by non-CSC dedifferentiation [11, 12], we sought to evaluate whether non-CSC dedifferentiation exists in lung cancer cells, and determine the role of MACC1 in non-CSCs dedifferentiation. We constructed a Tet-on-induced system to mediate MACC1 overexpression and depletion. After comparing the expression levels of MACC1 in common lung cancer cell lines (Fig. S1A), we used lentivirus infection to overexpress MACC1 in A549 cells with relatively low level (A549-pTRE3G-Tet3G-MACC1), and induced MACC1 depletion through short-hairpin RNA (shRNA) in NCI-H1299 cells with relatively high endogenous MACC1 expression abundance (H1299-pLKO-Teton-shMACC1). The efficiencies of MACC1 knockdown and overexpression were subsequently confirmed by western blotting (Fig. S1B, C). Then we purified ALDH^-^ cells sub-populations from both cell lines by fluorescence-activated cell sorting (FACS), cultured them for 7 days in the absence or presence of Dox to alter MACC1 expression levels. Afterwards we monitored the emergence of ALDH^+^ cells, which was characterized to possess stem-like properties (Fig. 2A). We indeed detected the conversion of ALDH^-^ cells into ALDH^+^ ones, and this process can be promoted or suppressed by Dox-induced MACC1 overexpression or knockdown (Fig. 2B–E).

To pinpoint the effect of MACC1 expression level alteration on the non-CSCs to CSCs conversion, we performed spheroid formation, colony formation and cell viability assays (cell counting kit-8, cck-8) in the ALDH^-^ cells after culturing with Dox or without for 7 days, as described in Fig. 2A. The ALDH^-^ cells which were deprived stem-like properties regained sphere formation ability after 7 days culture (Figs. 2F, I left panel), proving the existence of the non-CSCs to CSCs conversion process. Elevated or decreased MACC1 expression (Dox^+^) enhanced or impaired the sphere formation rate in vitro after 7 days of culture (Fig. 2F–K). Moreover, we also noticed that knocking down MACC1 (Dox^+^) significantly inhibited the malignant proliferation potential of H1299 ALDH^-^ cells (Fig. 2O–Q). In addition, the opposite result was confirmed by Dox-induced MACC1 overexpressing of A549 ALDH^-^ subpopulation after 7 days culture (Fig. 2L–N), supporting the role of MACC1 in modulating non-CSC-to-CSC conversions in lung cancer cell line.

### MACC1 silencing inhibited non-CSCs dedifferentiation of lung cancer in vivo by retracting tumorigenic potential and lung-CSCs-derived xenograft cells frequency

**Fig. 3:**
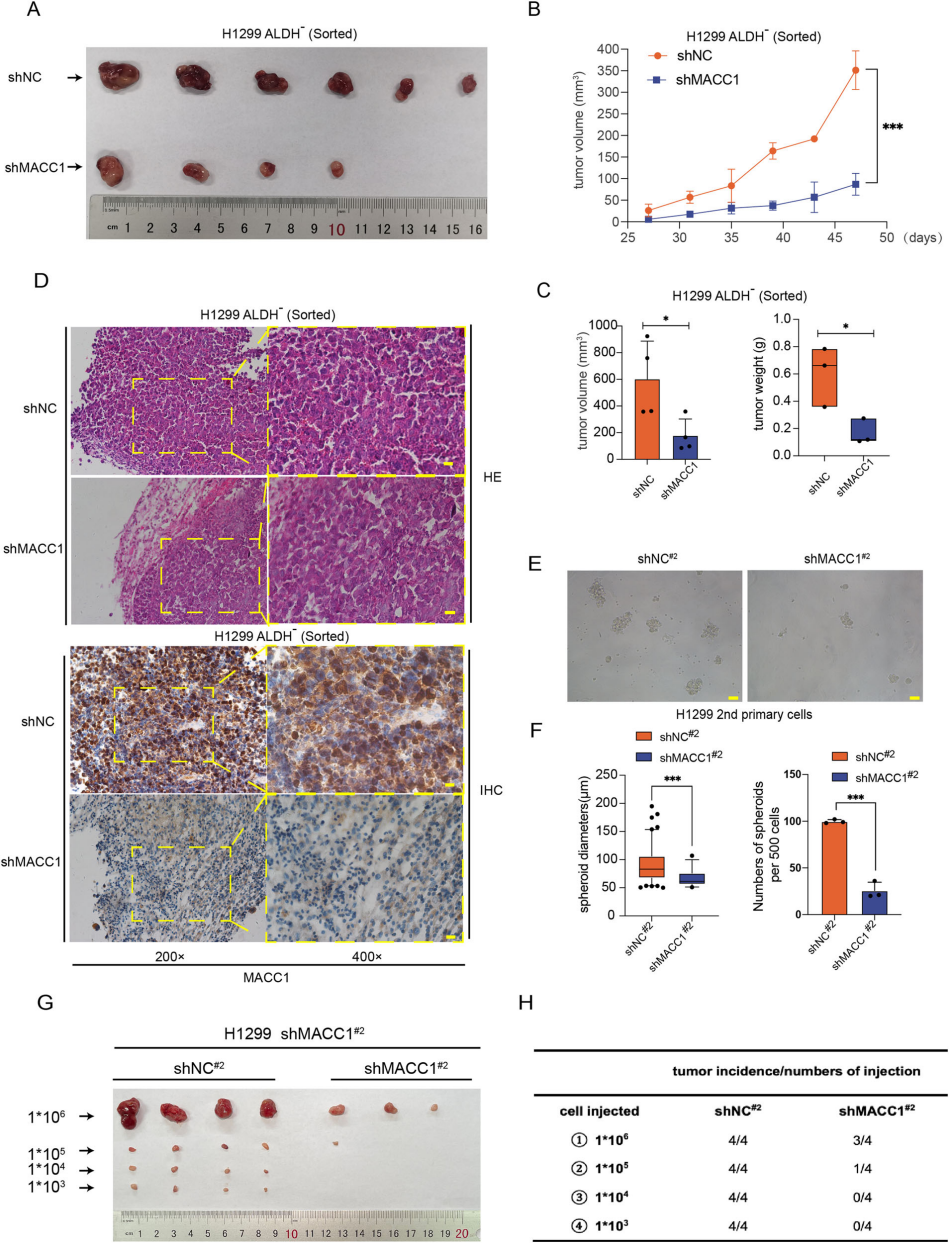
MACC1 silencing inhibited non-CSCs dedifferentiation of lung cancer in vivo by retracting tumorigenic potential and lung-CSCs-derived xenograft cells frequency. Immunodeficient mice (*n* = 6, 1 site per mouse) were subcutaneously inoculated with equal number of single cells in the absence or presence of Dox from H1299 ALDH^-^ (sorted) (1 × 10^6^ cells per mouse). Tumor xenografts were monitored for 8 weeks. Tumor volumes were monitored as described in Materials and methods, tumor images (**A**), growth curve (**B**), tumor volume and weights (**C**) were shown. *P < 0.05, two-tailed Student’s t-tests. Error bars: mean ± SD. Scale bars, 100 μm. H&E staining and immunohistochemical analysis of MACC1 protein expression (**D**) in tumor samples from H1299 ALDH^-^ (sorted). **E**, **F** Sphere formation ability of primary cells isolated from Dox absent and Dox present xenograft respectively. ***P < 0.001, two-tailed Student’s t-tests. Error bars: mean ± SD. Scale bars, 100 μm. **G** Limiting diluted numbers of isolated tumor cells were subcutaneously inoculated into immunodeficient mice (*n* = 4, 4 sites per mouse). **H** Serial transplantation frequencies were analyzed after 8 weeks.

To further inspect the role of MACC1 in non-CSCs dedifferentiation of lung cancer in vivo, we conducted tumor formation experiments in nude mice (BALB/c). As described in Fig. 2A, the sorted ALDH^-^ H1299 pLKO-tet-on shMACC1 cells which were lack tumorigenicity cultured in the presence and absence of Dox after 7 days, and were subcutaneously injected into 4-week-old nude mice with an equal number of cells (1 × 10^6^ per mouse, *n* = 6, see details in Materials and Methods section). We noticed that Dox absence group gained the tumorigenic potential after 7 days culture, demonstrating the non-CSCs dedifferentiation happened (Fig. 3A, top row). Particularly, MACC1 knockdown (Dox^+^) impaired the tumorigenic ability severely. we even did not notice the tumor formation in two mice during experiment period (Fig. 3A–C). MACC1 expression was obviously decreased in tumor tissues analyzed by IHC, confirming the silencing efficacy (Fig. 3D). Then we found A549 ALDH^-^ cells could also gain the tumorigenic potential after 7 days culture (Fig. S2A, top raw), dox-induced MACC1 overexpressing enhanced the tumorigenic potential significantly comparing with dox absent group (Fig. S2A–C). The overexpressing efficacy of MACC1 in the tumor tissues was confirmed by IHC analysis simultaneously (Fig. S2D).

Given the limiting dilution assay (LDA) is a well-established approach to accurately measure CSCs self-renewal capacity and tumorigenicity [35], we conducted the LDA to evaluate whether silence of MACC1 could impair non-CSCs dedifferentiation process by inhibiting tumorigenicity of xenograft-derived tumor cells. Primary cells were isolated from shNC (Dox absent) and shMACC1 (Dox present) xenograft respectively, referred as shNC#2^nd^ and shMACC1#2^nd^. Firstly, spheroid formation assay was employed, silence of MACC1 suppressed sphere quantity and size of primary cells from xenografts (Fig. 3E, F). Simultaneously, we inoculated subcutaneously in four different sites in each immunodeficiency (BALB/c) mice according to different dilution concentration gradients (Fig. 3G). After 8 weeks monitoring, the whole gradients of shNC#2^nd^ formed tumor effectively (100%), while the tumor formation rate of shMACC1#2^nd^ groups during the experimental period were 75, 25, 0 and 0% respectively (Fig. 3G, H).

Collectively, above results indicated that MACC1 is a pivotal regulator of non-CSCs dedifferentiation. Silencing of MACC1 was capable of delaying the conversion process of lung non-CSCs to CSCs, characterized by ALDH^-^ to ALDH^+^ cell conversions, as well as the non-tumorigenic to tumorigenic cells conversions.

### MACC1 is negatively correlated with KLF4 expression level

**Fig. 4:**
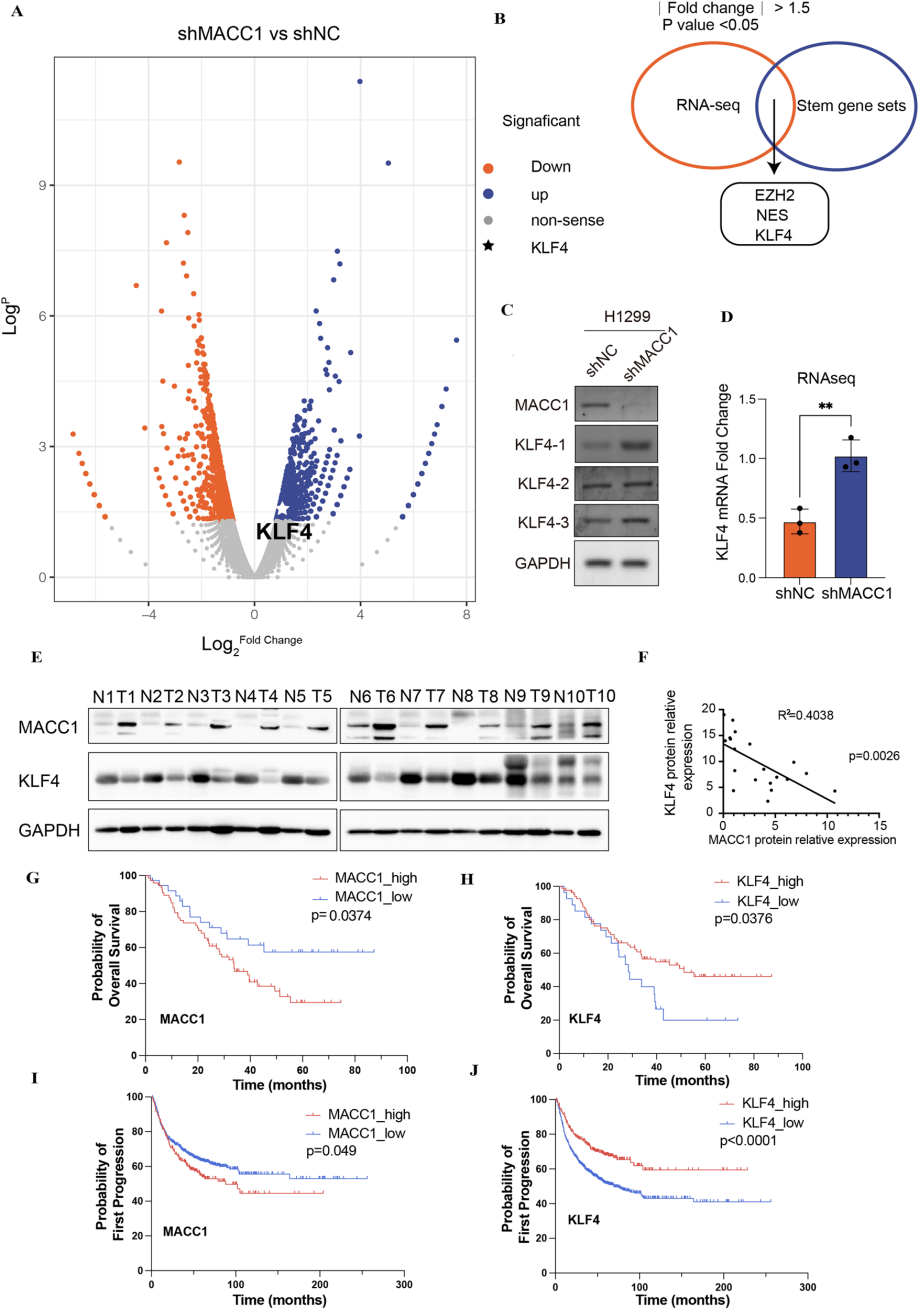
MACC1 is negatively correlated with KLF4 expression level. **A** Volcano plot display the RNA-seq genome expression differences profile between H1299 shNC and shMACC1 (**B**) Overlap of RNA-seq data (absolute value > 1.5-fold, *p* < 0.05) with stem gene set. **C**, **D** Validation of KLF4 gene expression by RT-PCR (*N* = 3). **P < 0.01, two-tailed Student’s t-tests. Error bars: mean ± SD. **E** MACC1 and KLF4 levels from ten paired NSCLC tumors (T) and normal (N) tissues were evaluated by western blots. **F** The correlation between the levels of MACC1 and KLF4 was plotted. **G**–**J** Kaplan–Meier regression curve analysis of the overall survival (GSE3141 from GEO database) and first progression (from https://kmplot.com/) of lung cancer patients with high or low expression of KLF4 and MACC1. *p* < 0.05 indicates a significant difference regarding survival time or first progression time between the two groups.

To better explore the potential mechanism of MACC1 mediated non-CSCs dedifferentiation, we performed RNA-seq of lung cancer cells (NCI-H1299) stably knocked down MACC1 and control. We screened out a series of up or down-regulated genes (absolute value > 1.5-fold, *p* < 0.05) and generated volcano plot (Fig. 4A). Then we compared the RNA-seq data with a stem-cell gene set [36], a total of three stemness-associated genes were overlapped out, including KLF4 (Fig. 4B). In order to verify the reliability of the sequencing results, we designed three pairs of primers at different sites to prevent off-target effects (Fig. 4C, D), we found KLF4 was upregulated after MACC1 knockdown. To assess the clinical relevance of MACC1 and KLF4 in tumorigenesis, we measured the protein levels of MACC1 and KLF4 in surgically collected paired NSCLC samples and adjacent normal lung tissues from ten patients. We found that all of the primary NSCLC specimens have a noticeable increase of MACC1 protein but decrease of KLF4 expression as compared to paired normal lung tissues, indicating that the level of KLF4 is negatively correlated to the level of MACC1 in patient samples (R^2^ = 0.4038, p = 0.0026) (Fig. 4E, F). Our previous studies also have shown that MACC1 is highly expressed in lung adenocarcinoma, overexpression of MACC1 is an independent risk factor for poor prognosis in patients with lung adenocarcinoma [24]. To validate the clinical significance of MACC1 and KLF4, we conducted the survival (Fig. 4G, H) and prognosis (Fig. 4I, J) analysis based on the GEO and Kaplan-Meier Plotter database. The results demonstrated that the patients with low MACC1 (OS *p* = 0.0374, FP *p* = 0.049) and high KLF4 (OS *p* = 0.0376, FP *p* < 0.0001) expression had significantly longer survival and first progression time than those with high MACC1 and low KLF4 expression.

Hence, we speculate that KLF4 may serve as a downstream regulatory target of MACC1 mediated non-CSCs dedifferentiation in lung cancer.

### KLF4 is a key effector of MACC1 mediated non-CSCs dedifferentiation in lung cancer

**Fig. 5:**
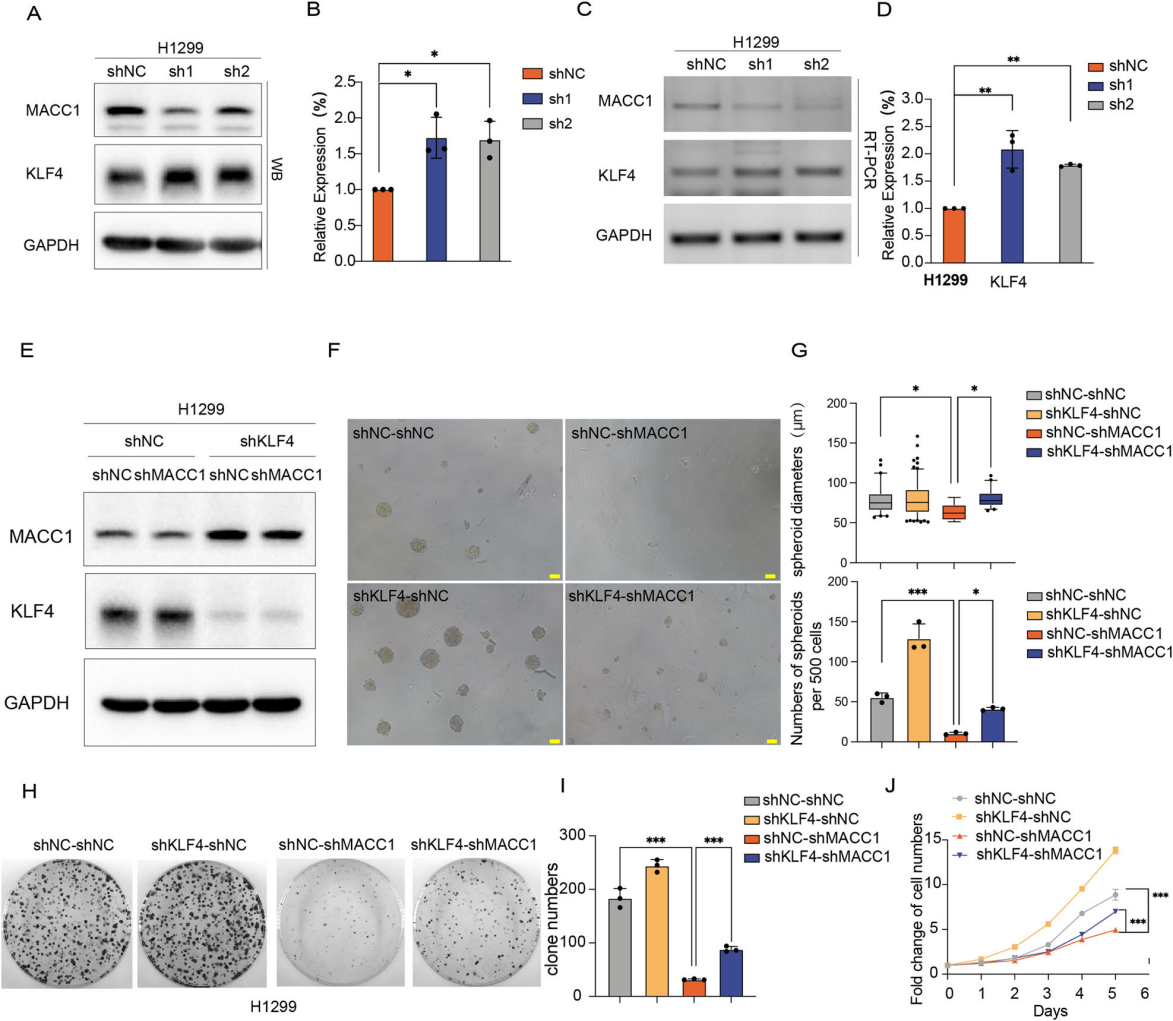
KLF4 is a key effector of MACC1 mediated non-CSCs dedifferentiation in lung cancer. **A**–**D** Validation of KLF4 of mRNA and protein level were examined following MACC1 silence in H1299 cell line. column graph displayed relative quantification of KLF4 mRNA and protein after three replicates, *P < 0.05, **P < 0.01. Error bars: mean ± SD. **E** shRNA-mediated KLF4 suppression in both H1299-shNC and H1299-shMACC1 cells. MACC1 and KLF4 expression levels were examined by western blot. Sphere-forming abilities (**F**, **G**), colony-forming ability (**H**, **I**) and cell viability (**J**) were compared. *P < 0.05, ***P < 0.001. Error bars: mean ± SD Scale bars, 100 μm. The comparison of data among the above groups was conducted using ANOVA.

We stably knocked down MACC1 in two lung cancer cell lines of lung cancer cells (H1299, H2170), and set up two short hairpin RNA knockdown sequences to prevent the off-target effect of si-RNA. The regulatory effect of MACC1 knockdown on KLF4 was detected at the protein and RNA level. KLF4 protein levels were significantly upregulated when MACC1 was knocked down in lung cancer cell lines H1299 and H2170 (Fig. 5A, B, Fig. S3A, B). Similarly, mRNA level of KLF4 was also significantly upregulated after knocking down MACC1 (Fig. 5C, D, Fig. S3C, D). To determine whether knockdown KLF4 in lung cancer cell lines could restore the stem-like properties loss caused by knockdown of MACC1, we conducted the rescue experiments by stably knocking down KLF4 in the shMACC1 stable cell line and control, described as shKLF4-shMACC1 and shKLF4-shNC, the expression efficiency was shown in Fig. 5E. Firstly, we performed spheroid formation assay in two lung cancer cell lines. Compared with the shNC-shNC group, diameter and number of spheres decreased significantly in the shNC-shMACC1 group. While on the basis of knocking down MACC1 and continuing to knock down KLF4, the diameter of tumor cell microspheres in the shKLF4-shMACC1 group was significantly enlarged, and the number of spheres was also partially restored (Fig. 5F, G, Fig. S3F, G). Similar results also obtained in colony formation and cell viability assay (cck-8), by knocking down KLF4 in the shMACC1 stable cell line, the proliferation ability and cell viability of lung cancer cells can be significantly restored (Fig. 5H, I, Fig. S3H, I). Taken together, these results indicated that KLF4 is a key effector for the MACC1 silence suppress stem-like properties in lung cancer.

### MACC1 ablation stabilizes KLF4 through the post-transcriptional level

**Fig. 6:**
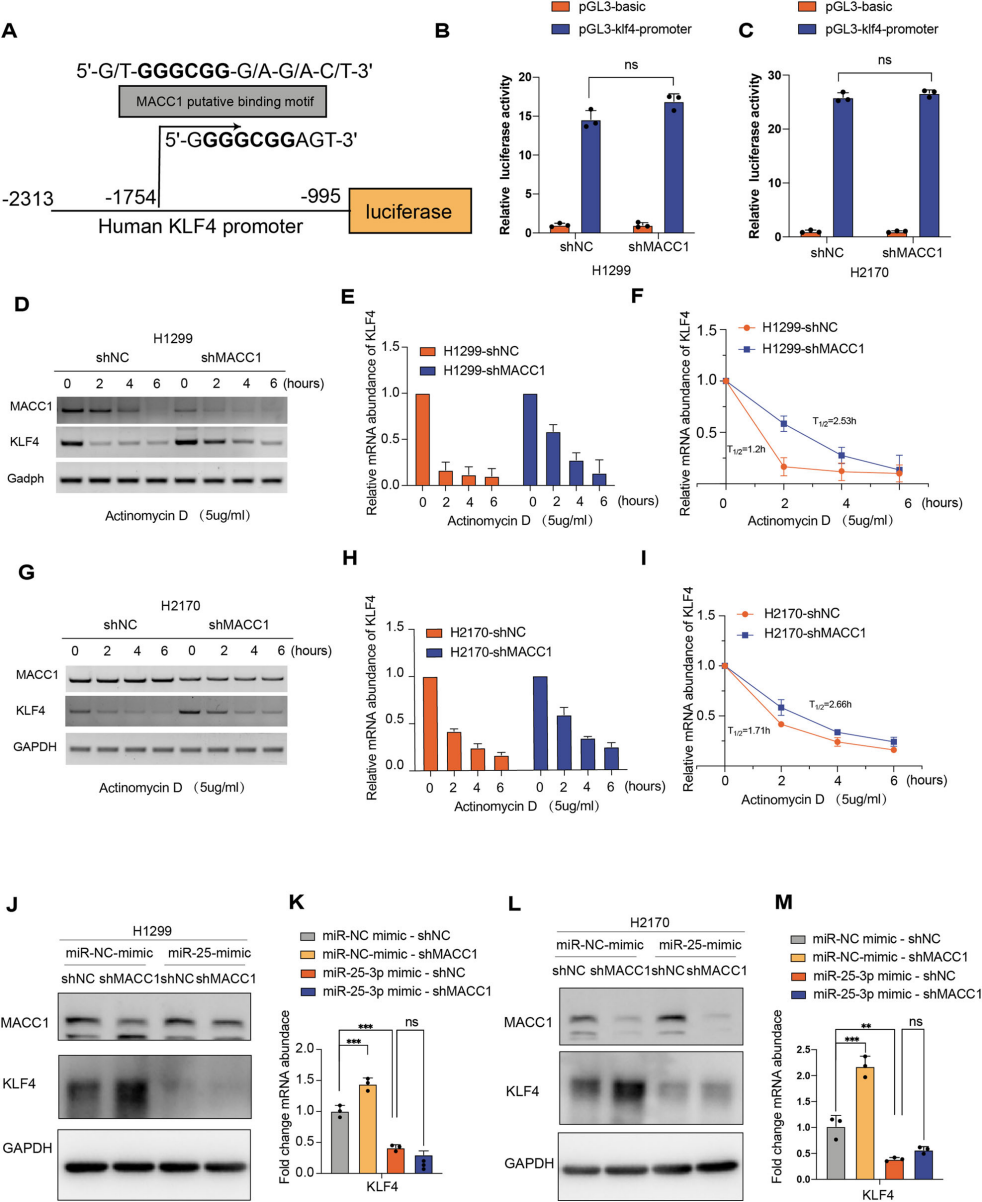
MACC1 silencing stabilizes KLF4 through the post-transcriptional level. **A** Schematic diagram of KLF4 promoter region including predicted binding sites for MACC1 was cloned into pGL3-basic plasmid, (**B**, **C**) and dual-luciferase assay was performed in H1299 and H2170 cells as described in Materials and Methods. Transcription activity was calculated as the ratio of Firefly luciferase activity (reporter) vs Renilla luciferase activity (control). Comparison was analyzed by two-tailed Student’s t-tests. ns=no significance, Error bars mean ± SD. **D**, **G** NCI-H1299 and NCI-H2170 cells were stably knockdown MACC1, actinomycin D (ActD, 5 μg/ml) was added for indicated time points (0, 2, 4 and 6 hours) before harvest. KLF4 mRNA levels were assessed by RT-PCR. MACC1 knockdown efficiencies were determined by RT–PCR analysis. **E**, **H** Relative fold of expression was calculated by normalizing mRNA level to that at 0 h (without ActD treatment) for both shNC and shMACC1 groups. Results were representative of three independent experiments. Error bars: mean ± SD. **F**, **I** Half-life of mRNA for each group was calculated and compared. The miR-25 activities were reconstituted by mimics. Relative mRNA abundance and protein level of KLF4 were examined by RT-qPCR assay and western blot., **P < 0.01, ***P < 0.001, AVOVA, ns=no significance, Error bars, mean ± SD.

To explore the molecular mechanism of KLF4 mRNA level up-regulation mediated by MACC1 silencing. First, we investigated the effect of MACC1 on the transcriptional level of KLF4 using dual-luciferase reporter gene system. MACC1 was identified as a transcription factor to control the Met promoter activity and expression [18], Met promoter fragment harbors key consensus motifs as known as Sp1 binding sites, including 5’-G/T-GGGCGG-G/A-G/A-C/T-3’ or 5’-G/T-G/A-GGCG-G/T-G/A- G/A-C/T-3’ [37]. Among them Sp1-1 binding site (5’-GGGCGG-3’) is of essential importance for the MACC1-induced Met promoter activity [38], the promoter fragment of KLF4 also harbors sp1-1 binding site coincidentally. Therefore, we speculate that MACC1 can regulate the transcription of KLF4 gene by binding sp1-1 motif. We cloned the fragment of KLF4 promoter (Fig. 6A) by PCR and inserted into luciferase reporter pGL3-basic plasmid through Sac I and Bgl III cloning sites. Unfortunately, the results of the dual-luciferase reporter showed that the luciferase firefly of the KLF4 gene did not change statistically significantly with the silencing of MACC1 in both H1299 and H2170 cell lines (Fig. 6B, C), suggesting that MACC1 does not control KLF4 promoter activity. Next, to identify whether MACC1 knockdown increases the stability of the KLF4 gene at post-transcriptional level, we treated the cells with 5 μg/ml actinomycin.D (ACTD) to block the de novo synthesis of mRNA, harvested the cells and extracted the total mRNA at four time points of 0, 2, 4, and 6 hours, and identified the mRNA expression level by RT-PCR (Fig. 6D, E and Fig. 6G, H). Compared with the control group, the half-life of the KLF4 gene in shMACC1 group was significantly prolonged from 1.2 h to 2.53 h in H1299 cell line (Fig. 6F). The half-life of KLF4 gene in shMACC1 group was significantly extended from 1.7 h to 2.66 h in H2170 cell line. The above results indicate that MACC1 silencing increased KLF4 gene stability, thereby making the expression level elevated.

**Fig. 7:**
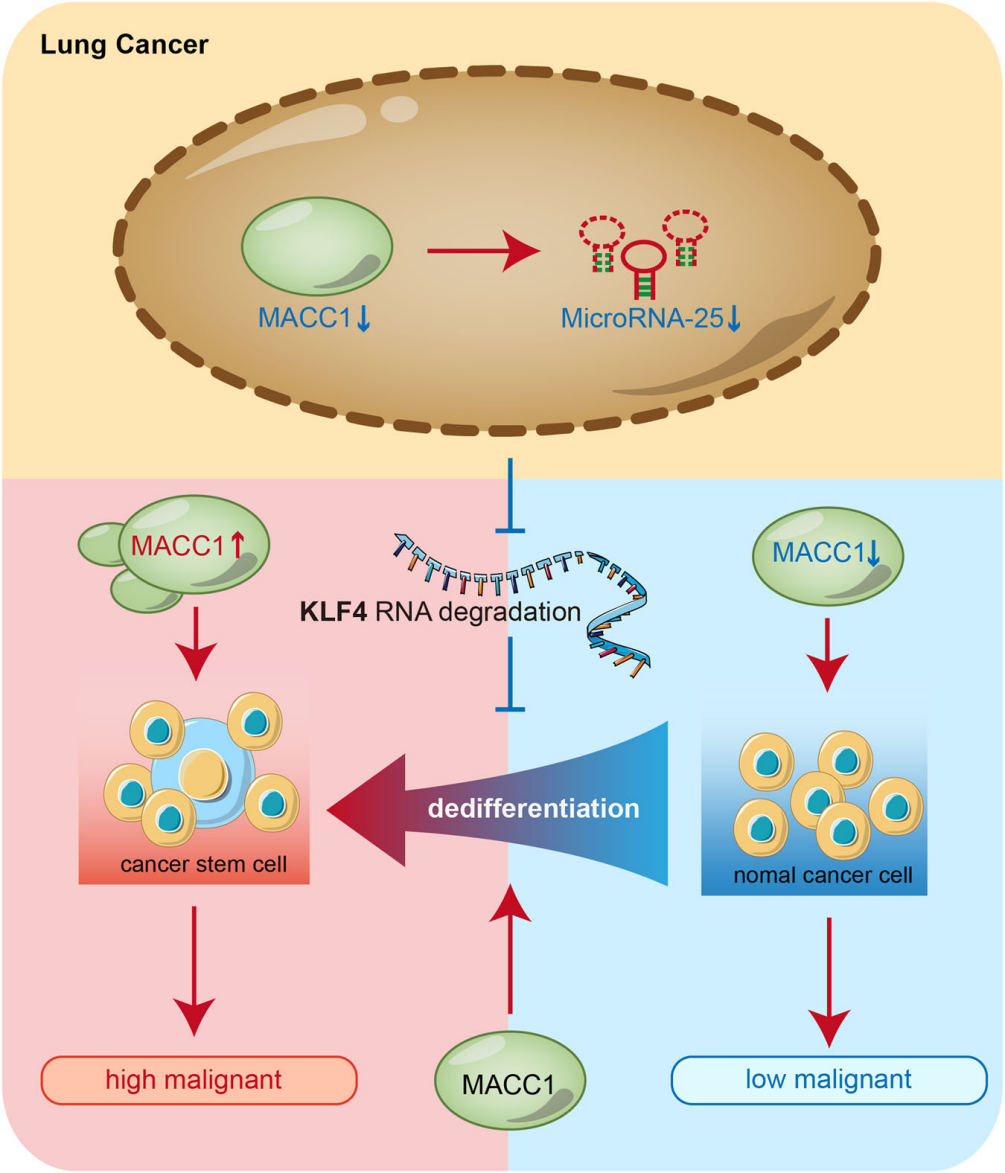
Schematic diagram depicting MACC1 influents the transition of non-CSC and CSC. In our system MACC1 silence delays KLF4 mRNA degradation through downregulating MicroRNA-25 in lung cancer cells, and interdicts the transition from non-CSC to CSC, and inhibits lung cancer cells malignant character accordingly.

Previous studies indicate that MicroRNA-25 (miR-25) directly target the conserved 3’UTR region of KLF4, thereby inhibiting the expression of KLF4 [39–41]. Surprisingly, we found that the expression level of mature miR-25, miR-25-3p was also down-regulated after MACC1 knockdown by RT-qPCR in lung cancer cell line, consistent with previous reports (Fig. S4A). To test whether MACC1 regulate KLF4 via miR-25 and to determine whether the reduced expression of miR-25 was responsible for MACC1 silence mediated stabilization of KLF4 mRNA, we performed rescue experiments by transfecting miR-25 mimics in MACC1 stably knockdown in lung cancer cells. The results demonstrated that reactivated miR-25-3p by the mimics abrogated the MACC1-mediated elevation of KLF4 at the mRNA and protein levels in two cancer cell lines (Fig. 6J–M). In conclusion, our findings unveiled MACC1 as a key regulator for conversation of non-CSCs to CSCs. Further evidence suggests that miR-25 plays an essential role in the regulation of KLF4 mRNA stability and expression by MACC1 silence (Fig. 7).

## Discussion

The CSC theory states that tumor growth is fueled by a small number of cancer stem cells that lurk within the cancer. In the past two decades an avalanche of reports on the identification of CSCs appeared in many common cancer types, including lung cancer [42–45]. CSCs contributes to most inevitable recurrence and drug resistance during lung cancer treatment, it inspires researchers to propose novel treatment concepts based on CSCs characteristic, e.g., eradicating them completely or making them transitional.

Multiple researches have provided evidence CSCs and non-CSCs are plastic and capable of interconversion under specific stimuli. Signaling factors within the tumor microenvironment can induce non-CSCs to acquire CSC properties. For example, in an inflammatory milieu, the activation of NF-κB signaling can contribute to the carcinogenic potential during the progression of colorectal cancer by inducing the dedifferentiation of non-stem cells [46]. It is suggested that hepatocyte growth factor (HGF), which is secreted by fibroblasts located in specific tumor microenvironments, increases WNT signaling and endows non-CSCs with self-renewal and tumorigenic capabilities [47]. The overexpression of EMT (epithelial-to-mesenchymal transition) transcription factors has been shown to not only drive a mesenchymal-migratory phenotype but also to enhance the tumor-initiating potential of cell lines [48, 49]. Depending on the expression of the EMT inducer ZEB1, basal breast cancer cells switch between non-CSC and CSC states [50]. It also has been illustrated that briefly expressing TWIST1 inclines mammary cells into a CSC-like state [51]. Epigenetic mechanisms such as DNA methylation, histone modification, etc. can also lead to changes in cell phenotype, transforming non-CSCs into CSCs, the ovarian CSC subpopulation may be preserved through cancer cell dedifferentiation, and DDB2 can prevent the transformation of non-CSCs into CSCs by suppressing ALDH1A1 transcription [11]. In the sum up, it is a dynamically reversible process from “adult” to “baby”, which is subversive compared to classical CSCs insight.

Therefore, illumination of key drivers and mechanism involved in the process is an urgent problem to be solved. We provide evidence in this study showing that non-CSCs dedifferentiation process also exists in lung cancer cells, and uncovered here for the first time that MACC1 was enriched in the lung CSCs subpopulations (Fig. 1). We used the flow cytometry sorting to purify the stem cell subpopulation represented by ALDH^+^ and the non-stem cell subpopulation represented by ALDH^-^. Hereby we observed that MACC1 was upregulated in parallel with the elevation of stemness markers Nanog and Sox2 in ALDH^+^ cell population. MACC1 was also upregulated in the stemness-enriched cell subsets of lung cancer according to the GEO database.

MACC1 was firstly discovered as an important transcriptional regulator of metastasis-related genes [18]. It was involved in various signaling pathways such as MAPK and Wnt. c-MET and SPON2, as transcriptional targets of MACC1, induced metastasis in xenograft and genetically modified mouse models, and is closely related to the metastasis of cancer patients [18, 52]. In recent years, a series of studies have shown that MACC1 is related to stemness. Within 5-FU-resistant cells, cell death was facilitated by MACC1 knockdown. Furthermore, sphere formation and the expression levels of pluripotent markers, including cluster of differentiation CD44, CD133 and Nanog were reduced due to MACC1 depletion [53]. Studies showed that overexpression of MACC1, CD44, Twist1, and LNM and TNM stages were independent predictors of prognosis in patients with colonic adenocarcinoma [54]. Indeed, our reports confirmed MACC1 was required for the transition from non-CSCs to CSCs (Figs. 2, 3 and Fig. S2).

Next, according to RNA-seq analysis, KLF4 was elevated by MACC1 silence. KLF4 transcription factor, a critical regulator of cell biology process, including cell cycles, apoptosis, cancer metastasis and cancer stem cells [55]. As to the function on stems cell or CSCs, recent studies have shown that KLF4, as a negative regulator of CD44, has the function of inhibiting the characteristics of cancer stem cells in pancreatic ductal carcinoma [14]. Consistently, restoration of KLF4 expression alone was sufficient to shut off cell motility and eliminate the progenitor-like population of lung cancer cells [15]. Therefore, KLF4 can be used as a negative regulator of cancer stem cells in lung cancer, and it is also consistent with the result of knocking down MACC1 to inhibit the dedifferentiation of non-CSCs in lung cancer by up-regulating the expression of KLF4 (Figs. 4, 5 and Fig. S3). In an effort to explore the mechanism underlying KLF4 elevated mediated by MACC1 silencing, we found MACC1 silence stabilized KLF4 mRNA by post-transcriptional level rather than affected its transcriptional activity. Previous research demonstrated miR-25 interacted with KLF4 and downregulated expression level by binding to its 3’-UTR region [41]. We found miR-25 as an essential effector in the regulation of KLF4 mediated by MACC1 silence. Nevertheless, the exact mechanism accounting for the MACC1 mediated repression of miR-25 requires further evaluation.

To sum up, this work conducted in-depth research on the dedifferentiation of non-CSCs in lung cancer, and confirmed that MACC1 played a vital role in it through post-transcriptional regulation, providing a potential therapeutic target and theoretical basis for the precise treatment of cancer stem cells in lung cancer.

## Materials and Methods

### Cell culture

Human lung cancer cell lines (A549, NCI-H1299, NCI-H2170, NCI-H358, NCI-H460 and NCI-H446), normal human lung fibroblast cells (MRC-5) and human embryonic kidney HEK293T cell line were purchased from American Type Culture Collection (ATCC). Cell lines were tested and authenticated, and were not cultured continuously for more than 3 months. Each cell line was cultured in its standard medium as recommended by ATCC. A549 cells were cultured in F-12K Medium (Hyclone) supplemented with 10% FBS (Gibco). HEK293T cells were cultured in DMEM Medium (Life Technologies) supplemented with 10% FBS (Gibco). NCI-H1299, NCI-H2170, NCI-H358, NCI-H460 and NCI-H446 cells were cultured in RPMI1640 Medium supplemented with 10% FBS (Gibco). All cells were maintained at 37 °C in a humidified 5% CO_2_ atmosphere.

### Plasmid construction

Full length of MACC1 fragment was cloned from human genome cDNA and ligated into pLVX-pTRE3G vector. The primers were as follows: 5’ NotI, 5’-CACGCGGCCGCTATGCTAATCACTGAAAGAAAACATT; 3’ MluI, 5-CACACGCGTCTATACTTCCTCAGAAGTGGAGAAT. The KLF4 promoter fragment was cloned from human genomic DNA and ligated into pGL3-basic vector (Promega) upstream of the luciferase reporter. Primers were as follows: 5’ SacI, 5’-CACGAGCTCAAGAATGCTTCTGTGGTCGG; 3’ BglII, 5’-CACAGATCTTTCTCTCTGGTCGGGAAACT. The full-length KLF4 plasmid was kindly provided by Dr. Chuan-Chun Han (Institute of Cancer Stem Cell, Dalian Medical University, Dalian, 116044, China). The shRNA targeting MACC1(1#, 2#, 3#) was ligated into pLKO-tet-on vector and pLKO.1 vector (Addgene) in following sequence: shMACC1-1#, TGCCTTGATTTGAATACA; shMACC1-2#, CTGCCACCATTTGGGATT. shMACC1-3#, GAGTTAGTCGCACGTCTC. The shRNA targeting KLF4 was ligated into pLKO.1 vector (Addgene) in following sequence: TTGGTGAGTCTTGGTTCTAA. All plasmids were verified by sequencing.

### Lentivirus preparation and transfection

Lentivirus was produced in HEK293T cells using the 2nd generation packaging system plasmids psPAX2 (Addgene) and pMD2.G (Addgene). The cells were transfected using lipofectamine plus (Sagecreation) according to the manufacturer’s instructions. Supernatant was collected after 48 h transfection by centrifugation to remove any cellular debris. The resulting viral particles were used to infect the target cell line. The stably integrated cells were selected with 2 μg/ml of puromycin (Sigma-Aldrich).

To generate stable cell line expressing MACC1 on doxycycline (Dox) induction, we used the response vector pTRE3G with HA tagged, regulator vector pCMV-Tet3G and A549 cells. The full-length MACC1 was cloned into the response vector, packaged both vectors into lentivirus respectively, and infected A549 cells in 1:1 ratio. The stably integrated cells were selected with 5 μg/ml of puromycin and 800 μg/ml of G418 at 2 days after infection for 7 days to obtain individual colonies. To generate stable cell line knocking down MACC1 on doxycycline, we packaged pLkO tet-on-shMACC1#1, pLkO tet-on-shMACC1#2 and pLKO tet-on empty vector into lentivirus, and infected H1299 cell line, the stably integrated cells were selected with 2 μg/ml of puromycin for 7 days. The inductions were carried out by adding doxycycline to a final concentration of 2 μg/ml. The expression of transgenes was confirmed by western blots before further analysis.

### Dual-luciferase reporter assay

The pGL3basic (Promega, USA) was used as control promoter and pRSV-Renilla luciferase expression vector (Promega, USA) was used to monitor transfection efficiency. H1299 or H2170 MACC1 knockdown stable cell lines were transfected with KLF4 promoter-driven luciferase vector pGL3-KLF4 or control pGL3-Basic luciferase vector using lipofectamine plus (Sagecreation) according to the manufacturer’s instruction. Cells Luciferase activity was measured using the Dual-Luciferase Reporter Assay system (Promega) according to the manufacturer’s instructions 24 h later. The Firefly luciferase activity was normalized to Renilla levels and is shown relative to control conditions.

### RT-PCR and quantitative RT-PCR validation

Total RNA was extracted from cells according to the manufacturer’s instructions. Genome DNAs were removed by 30 min DNase I (Takara) treatment at 37 °C and then heat inactivation using 50 nM EDTA. Total RNA (2 µg) was then reverse-transcribed with PrimeScript RT reagent kit (Takara) with random primer, and 1 µl of the cDNA was used as the template for PCR amplification. RT-PCR products were separated on 2% gels. The amount of each was measured by comparison of the integrated optical density of detected bands using the Image J. Real-time quantitative PCR was performed using Takara SYBR II kit on system according to the manufacturer’s instructions. The primer sequences see the supplementary table.

### RNA sequencing analysis

We constructed H1299 shNC and shMACC1 stable cell lines, and then total RNAs were extracted using Trizol. We used the Illumina TruSeq Total RNA Sample Prep kits to purify poly-adenylated RNA (not exceeding 2 µg) after RNase free DNase digested as per manufactures instruction. Cytoplasmic rRNA was removed by the RiboZero Human. Prior to generation of cDNA library with bar coded ends the mRNA purified were further analyzed using Bio-analyzer (Agilent Technologies). The protocol employed to prepare RNA-seq libraries was based on the use of Illumina TruSeq Total RNA Sample Prep kits. The cluster generation and sequencing were carried out by standard procedures in HiSeq 2000 Illumina platform with pair-end 150 bp (PE 150) sequencing strategy.

### RNA stability assay

H1299, H2170 MACC1 knockdown stable cells and control cells were treated with 5 µg/ml actinomycin D (ActD, Sigma, CA1201) and collected at the indicated time points. The total RNAs were extracted by Trizol reagent (Invitrogen) and analyzed by RT-PCR.

### Immunoblotting

Cells were harvested with RIPA lysis buffer containing 1 mM Cocktail and 1 mM PMSF. Cell debris was removed by centrifugation. The protein samples (20 µg) were boiled at 100°C for 5 min in 1× SDS sample buffer and fractionated by 10% SDS-PAGE and transferred to PVDF membrane. The membranes were gently rinsed with TBST and then incubated for 1 hour at room temperature in a solution containing 5% fat-free milk. The following antibodies were used: MACC1 (Abcam, Ab106579, 1:1000), KLF4 (Proteintech, 17402-1-AP, 1:1000), Gapdh (Sigma-Aldrich, SAB1405848, 1:5000), P21 (Cell Signaling Technology, 12D1, 1:1000), P27 (Cell Signaling Technology, D69C12, 1:1000), Ki-67 (Cell Signaling Technology, 9449 s, 1:1000), HA-Tag (Convance, mms-101p-1000, 1:1000). Secondary antibody was used: Anti-mouse or Anti-rabbit IgG, 1:5000 dilution (Cell Signaling Technology). Bound antibodies were visualized with enhanced chemiluminescence (Tanon) by MiniChmei Chemiluminescence imager (SAGECREATION, Beijing).

### ALDEFLUOR assay and flow cytometric analysis & sorting

The ALDEFLUOR^TM^ kit (Stem Cell Technologies, Catalog #01700) was used to identify the cells expressing ALDH activity for sorting and analysis. Cells (2 × 10^5^) were incubated in assay buffer containing ALDH substrate BODIPY-aminoacetaldehyde (BAAA). As negative control, an aliquot of the ALDH substrated treated cells were immediately quenched with specific ALDH inhibitor, DEAB. Both tubes were incubated for 45 min at 37 °C. After incubation, cells were centrifuged and the pellets were resuspended with 500 μl PBS and store the cells at 4 °C. The result in fluorescence intensity of ALDH^high^ cells and ALDH^low^ cells was analyzed by flow cytometer (Beckman Coulter, CytoFLEX). For flow cytometric sorting, cells were washed twice with PBS and resuspended in PBS and were performed on a FACS flow cytometer (Beckman).

### Sphere formation assay

Sphere formation was performed in 96-wells ultra-low attachment plates (Corning) with DMEM/F-12 (Gibco) medium supplemented with 2% B27, 20 ng/ml EGF, and 20 ng/ml bFGF. Basal medium containing methylcellulose gel matrix (2%, M0512, Sigma-Aldrich, US) was used to prevent reaggregation of the cells, as described previously [56]. A549, NCI-H1299 and NCI-H2170 cells were seeded at a density of around 2 cells/μl and cultured at 37 °C with 5% CO_2_ After 9–14 days, spheres greater than 50 μm diameter were counted at ×40 magnification (Olympus) and analyzed using Image pro plus 6.0 software (Media Cybernetics).

### Colony formation assay

MACC1 stable knockdown H1299 or H2170 cells and MACC1 stable over-expressing cells were seeded in 60 mm dishes (500 cells per dish) and incubated at 37 °C, 5% CO_2_ in humidified incubator for 10 days or two weeks (updated with fresh medium every 3 days). Each treatment was carried out in triplicate. Colonies were fixed with 4% paraformaldehyde and stained with crystal violet solution.

### Cell viability assay

We determined cell viability by cell counting kit-8 assay. (CCK-8, APExBIO) Cells were added to a 96-well plate (1000 cells/well) and cultured overnight until cells were adherent, CCK-8 was added to the cells according to the manual and was incubated for 2 h. Then, the absorbance value at OD450 was measured.

### Xenograft mouse model

Experimental procedures regarding the animal study have been subject to rigorous evaluation and received ethical clearance from the Animal Experimental Ethics Committee of Dalian Medical University (Approval No. AEE21015). 4-week-old BALB/c nude mice were purchased from Vital River Laboratories (VRL, Beijing) for in vivo tumorigenicity study. Nude mice were randomly allocated into each group, and were injected subcutaneously with 1 × 10^6^ A549 cells stably Dox-induced overexpressing MACC1 (*n* = 6), NCI-H1299 cells stably knocking down MACC1 (*n* = 6). Doxycycline was administered in drinking water (dilute to 2 mg/ml in 5% sucrose) and the bottles were covered with aluminum foil to prevent degradation by light, and change the water every three days. Six mice were used for each group. The size of the tumor was determined by vernier caliper measurement of the subcutaneous tumor mass every 3 days. Mice were raised in the following 8 weeks. Nude mice died unexpectedly or tumor weight exceeded 10% of their body weight were excluded. At the end of measurement, all mice were euthanized, and tumors were excised for further analysis. Tumor volume was calculated as V = 1/2 (long diameter) × (short diameter) [2]. For all data points, five independent measurements were performed and means were used for calculation.

For limiting dilution assays, cells were serially diluted from 1 × 10^6^ to 1 × 10^3^ cells/100 μl in PBS containing 50% Matrigel (BD Biosciences), followed by subcutaneous injection into nude mice. Four mice were randomly allocated in each group. Tumor formation was examined once every 3 days, and the total observation period was 4 weeks. Solid tumor size measurement method as stated above.

### Primary cell isolation and culture

The mouse tumor samples were chopped into small pieces, and incubated in fresh complete medium with 5 mg/ml collagenase I solution with 0.2 mg/ml hyaluronic acid in shaker at 37 °C for 3 h with 220 r/min. After incubation, the suspensions were passed through 70 μm (FALCON, REF352350) pore filter and centrifuge at 1000 rpm for 5 min. The pellet was resuspended in the fresh DMEM containing 10% FBS and plated into 60 mm cell culture plates.

### Immunohistochemistry

Resected xenograft tumor tissues were fixed with 4% paraformaldehyde before embedded in paraffin. Prepared tissue sections (4 μm) were deparaffinized with xylene and rehydrated with graded alcohol. Tissue sections were then treated with blocking solution (ZSGB-Bio) for 30 min at room temperature, before primary antibody incubation overnight at 4 °C, followed by secondary antibody incubation for 1 h at room temperature. Tissue samples were stained using a peroxidase IHC assay kit (ZSGB-Bio, Beijing) according to the manufacturer’s instructions. Mounted sections were examined by light microscopy (Leica) and acquired images were analyzed with Image-Pro Plus software (version 6.0).

### Clinical tissues samples collection

Fresh lung cancer tissues and adjacent normal tissues were continuous enrolled from patients with pathologically and clinically confirmed lung carcinomas. All of tissue specimens were kept in liquid nitrogen and sectioned for protein extraction. The use of the clinical samples in this study was approved by the Ethics Committee of the first affiliated hospital of Dalian Medical University (PJ-KS-KY-2021-94). All human tumor tissue samples were acquired with the written informed consent of patients or their guardians before involvement in the research.

### Statistics

Statistical analyses were performed by prim 9.5.1 (GraphPad Software, San Diego, CA, USA). Biologically repeated experiments (*n* = 3) were carried out to allow statistical analysis. Results were illustrated by mean ± standard error of the mean (SE). Two-tailed Student’s t-test was performed to evaluate statistical differences between groups, one-way ANOVA was employed to compare data among multiple groups, post-hoc comparisons among groups were made using the Tukey’s HSD procedure. with calculated *p*-values of less than 0.05 deemed as significant differences. Overall survival and First Progression were assessed using the Kaplan-Meier method, and differences between survival curves were evaluated using the log-rank test. No blinding was used. Sample sizes were selected without predetermined effect size.

## Supplementary information

supplementary figure and legends

primers sequence

Full and uncropped western blots

## Data Availability

All data generated in this study are available from corresponding author on reasonable request.
