# Supplementary material for: MACC1 ablation suppresses the dedifferentiation process of non-CSCs in lung cancer through stabilizing KLF4
Source: Cell Death Discov. 2024 Dec 18;10:494. doi: 10.1038/s41420-024-02256-0 (PMC11655558; doi:10.1038/s41420-024-02256-0)
Supplement: Supplementary file 1 — supplementary figure and legends [file 41420_2024_2256_MOESM1_ESM.docx]

**
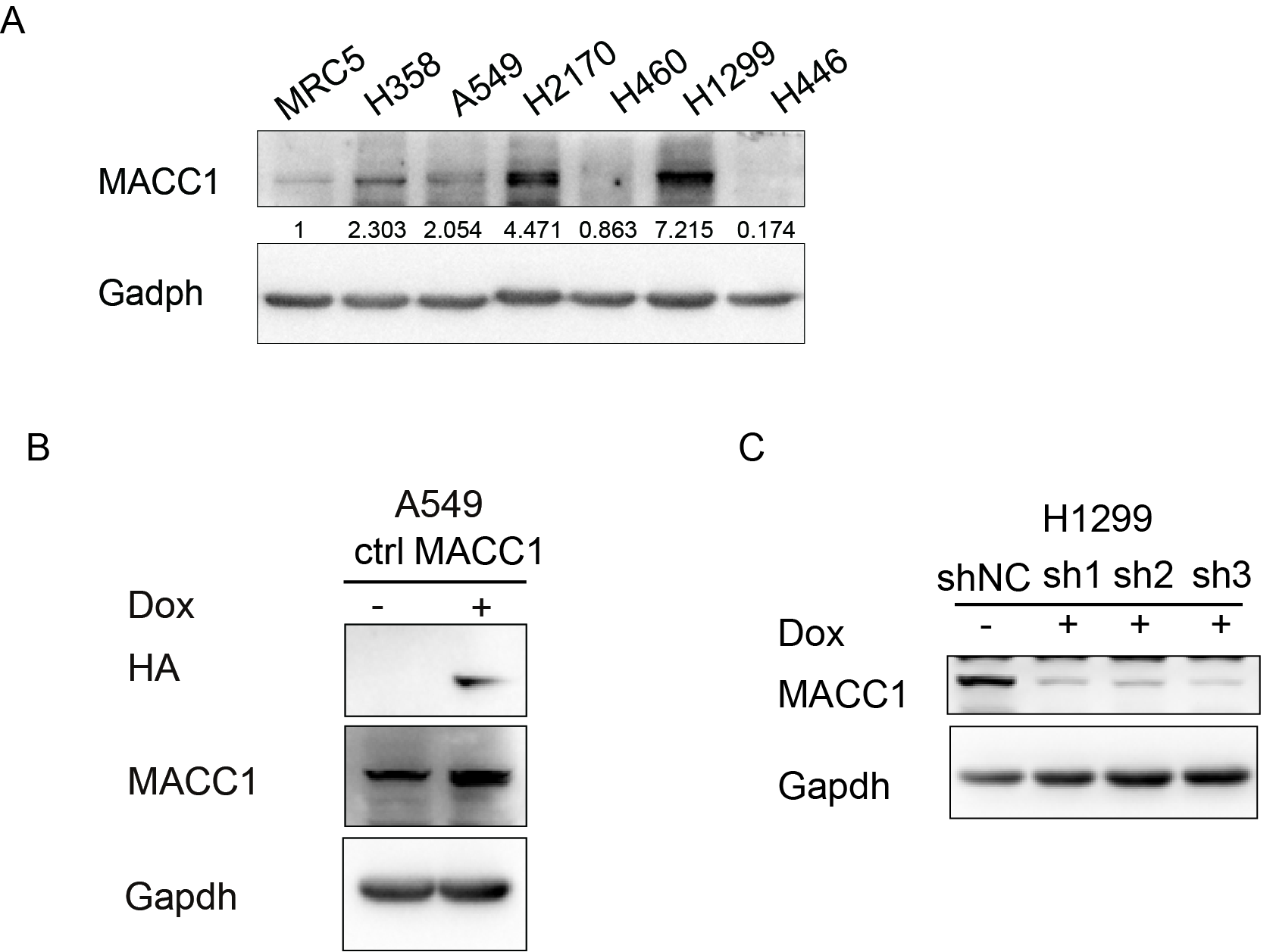
**

**Fig. S1** (A) Expression of MACC1 was examined by western blot in lung cancer cell lines MRC5 H358 A549 H2170 H460 H1299 and H446. (B-C) efficiency for MACC1 overexpression or Knockdown in A549 (Dox induced MACC1 overexpression) or H1299 (Dox induced MACC1 knockdown) were detected by western blot.


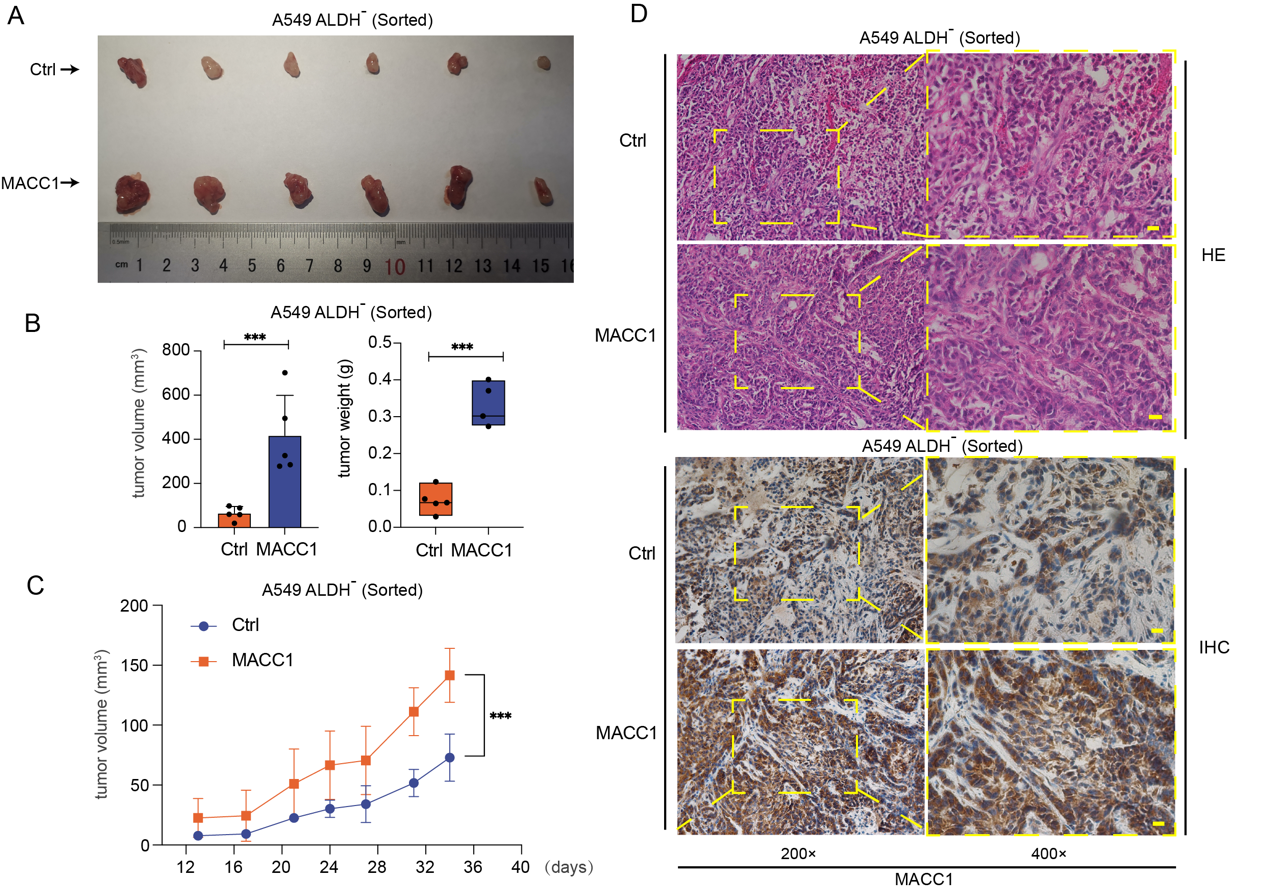


**Fig. S2** immunodeficient mice (n=6, 1site per mouse) were subcutaneously inoculated with equal number of single cells in the absence or presence of Dox from A549 ALDH^-^ (sorted) (1 × 10^6^ cells per mouse). Tumor xenografts were monitored for 8 weeks. Tumor volumes were monitored as described in **Materials and methods,** tumor images (A), growth curve (B), tumor volume and weights (C) were shown. ***P<0.001, two-tailed Student’s t-tests. Error bars: mean ± SD. Scale bars, 100 μm.

H&E staining and immunohistochemical analysis of MACC1 protein expression (D) in tumor samples from A549 ALDH^-^ (sorted).


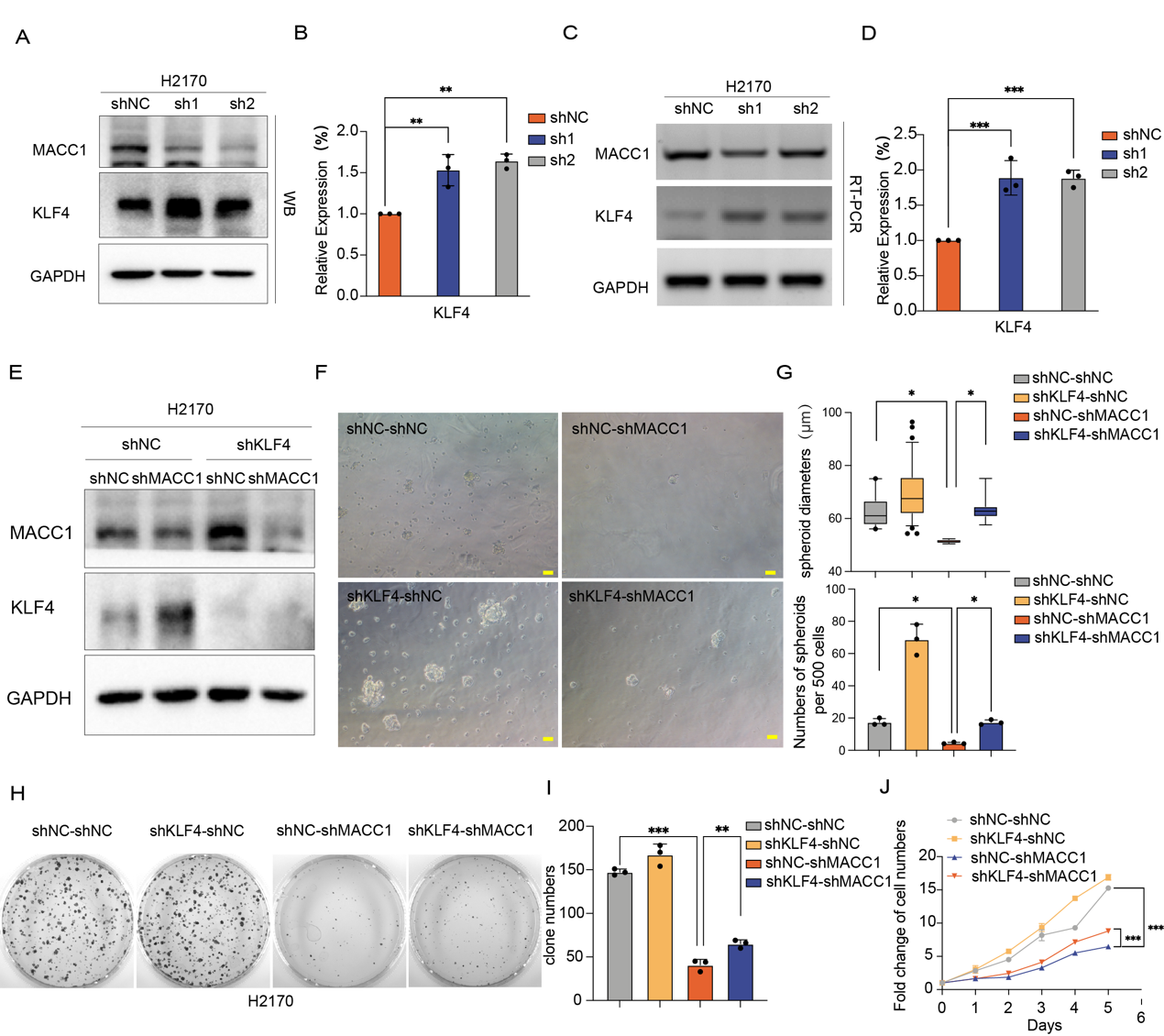


**Fig. S3** (A-D) Validation of KLF4 of mRNA and protein level were examined following MACC1 silence in NCI-H2170 cell line. column graph displayed relative quantification of KLF4 mRNA and protein after three replicates, **P<0.01, ***P<0.001. Error bars: mean ± SD. (E) shRNA-mediated KLF4 suppression in both H2170-shNC and H2170-shMACC1 cells. MACC1 and KLF4 expression levels were examined by western blot. And sphere-forming abilities (F and G), colony-forming ability (H and I) and cell viability (J) were compared. *P<0.05, **P<0.01, ***P<0.001. Error bars: mean ± SD Scale bars, 100 μm. The comparison of data among the above groups was conducted using ANOVA.

**
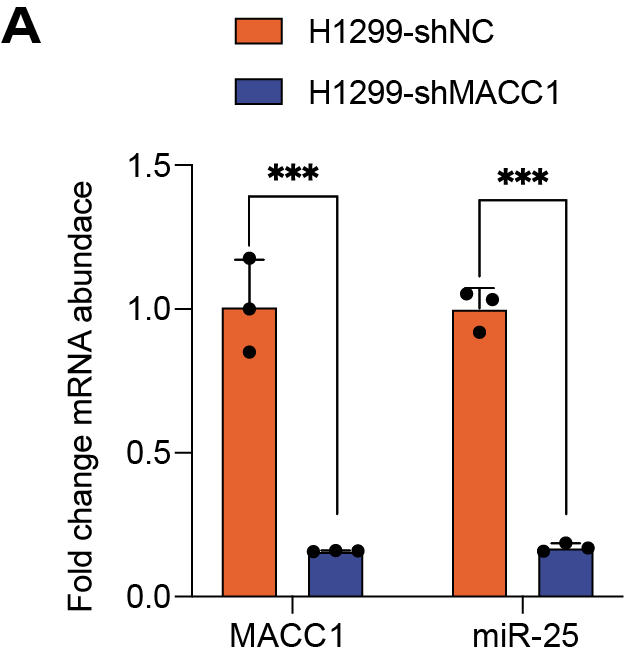
**

**Fig. S4** Expression of the miR-25 after MACC1 silence in H1299 stable cell line was analyzed by RT-qPCR assay (U6 was used as an internal control). Error bars represented mean ±SD. Comparison was analyzed by two-tailed Student’s t-tests. ***P<0.001
