# Supplementary material for: MACC1 ablation suppresses the dedifferentiation process of non-CSCs in lung cancer through stabilizing KLF4
Source: Cell Death Discov. 2024 Dec 18;10:494. doi: 10.1038/s41420-024-02256-0 (PMC11655558; doi:10.1038/s41420-024-02256-0)
Supplement: Supplementary file 2 — primers sequence [file 41420_2024_2256_MOESM2_ESM.docx]

| Name | sense sequence 5'-3' | antisense sequence 5'-3' |
| --- | --- | --- |
| MACC1 | TCTCCTCGGGGAAGGTAAGG; | TCTTGGATGAGACGTGCGAC; |
| KLF4-1 | ACCCTGGGTCTTGAGGAAGT; | GGCATGAGCTCTTGGTAATGGA; |
| KLF4-2 | GATGATGCTCACCCCACCTT; | ATGCTCGGTCGCATTTTTGG; |
| KLF4-3 | ATGCTCGGTCGCATTTTTGG; | GTTCATCTGAGCGGGCGAAT; |

primers for RT-PCR

primers for RT-qPCR

| Name | sense sequence 5'-3' | antisense sequence 5'-3' |
| --- | --- | --- |
| U6  Mir25  KLF4 | CTCGCTTCGGCAGCACA; GCAGCATTGCACTTGTCTCG; GGGCCCAATTACCCATCCTT | AACGCTTCACGAATTTGCGT;  AGTGCAGGGTCCGAGGTATTC;  CAGCCCGAGCTACAAATCCC |

primers for Mir25 reverse transcription

| Name | Sequence 5'-3' |
| --- | --- |
| Mir25 | GTCGTATCCAGTGCAGGGTCCGAGGTATTCGCACTGGATACGACTCAGAC |
