# Supplementary material for: MACC1 ablation suppresses the dedifferentiation process of non-CSCs in lung cancer through stabilizing KLF4
Source: Cell Death Discov. 2024 Dec 18;10:494. doi: 10.1038/s41420-024-02256-0 (PMC11655558; doi:10.1038/s41420-024-02256-0)

Figure 1

Figure 1C-MACC1

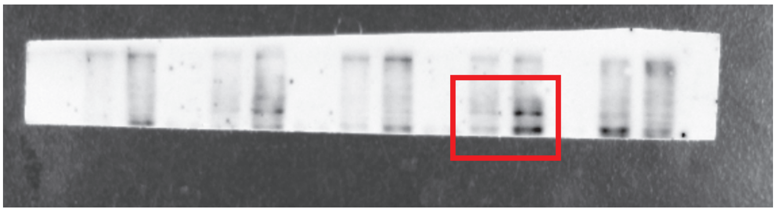

Figure 1C-Sox2

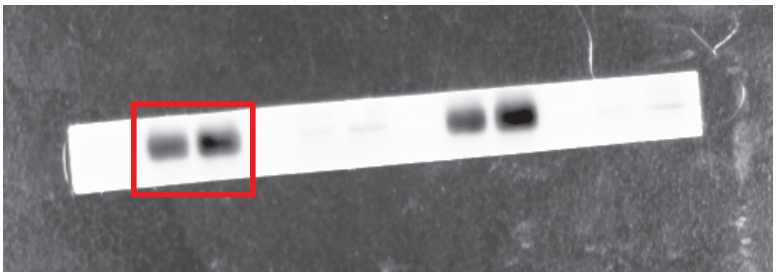

Figure 1C-nanog

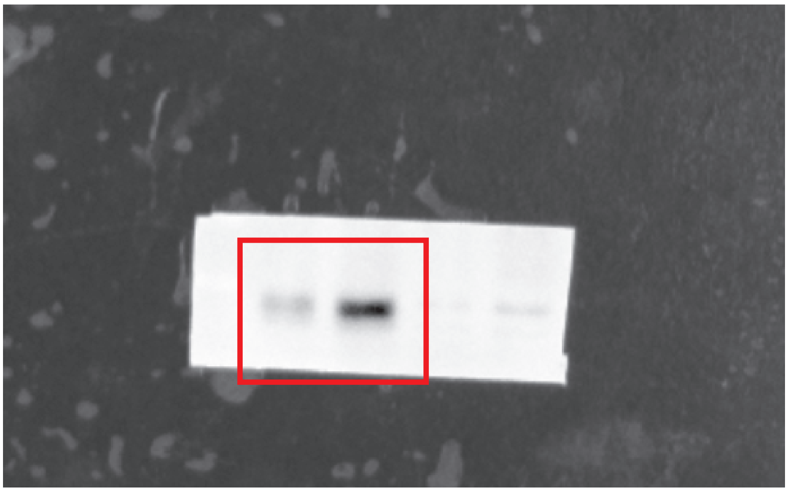

Figure 1C-GAPDH

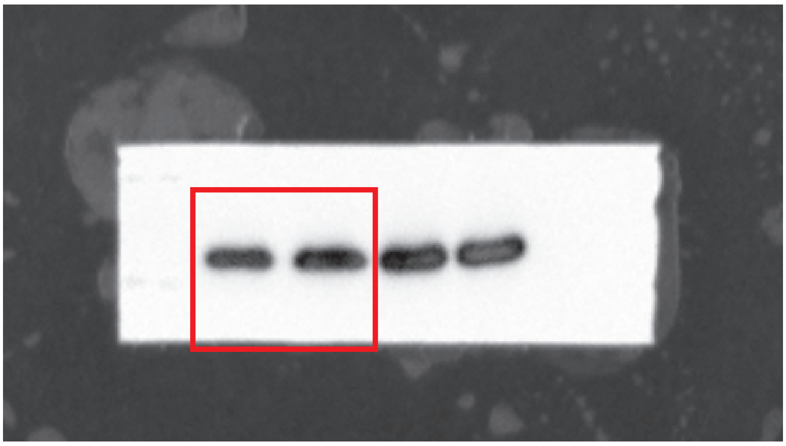

Figure 1E-MACC1

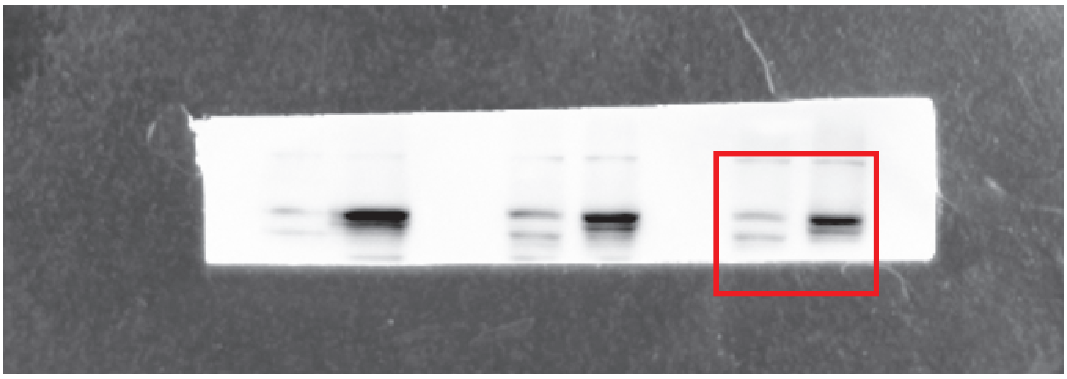

Figure 1E-Sox2

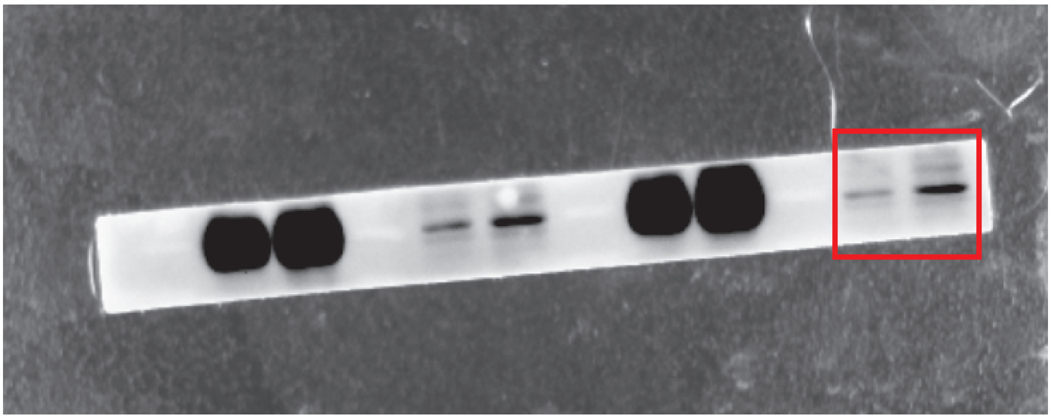

Figure 1E-Nanog

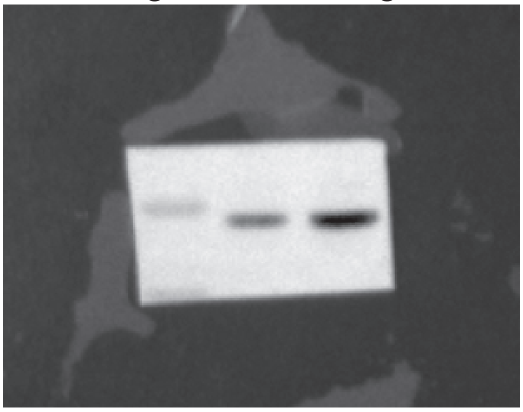

Figure 1E-GAPDH

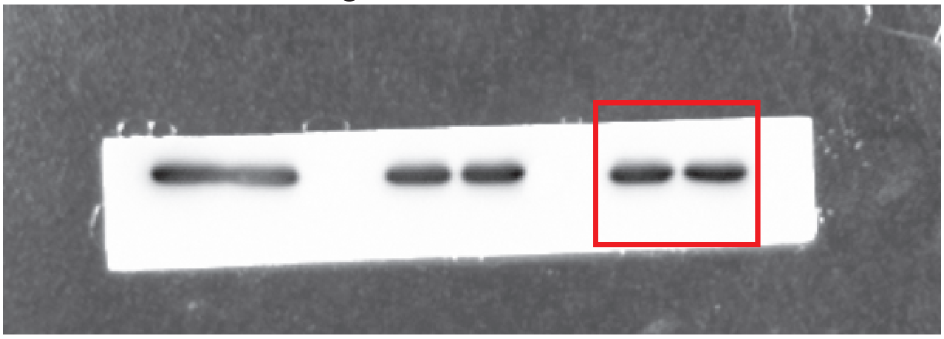

Figure 1G-MACC1

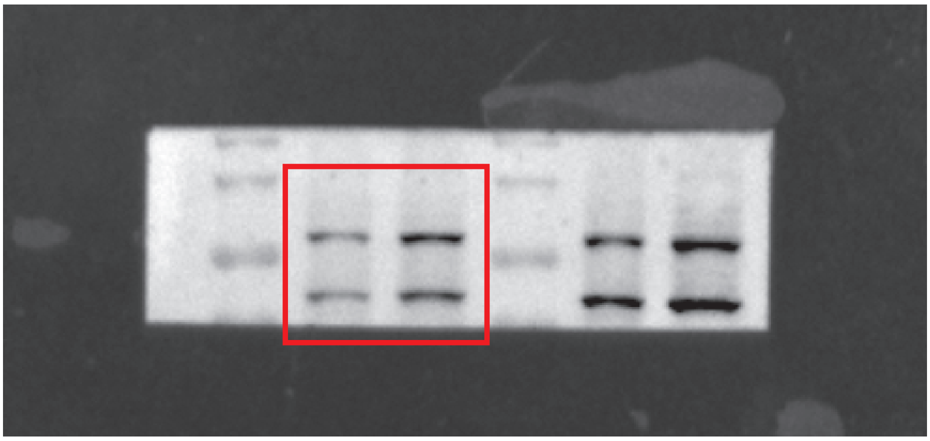

Figure 1G-Sox2

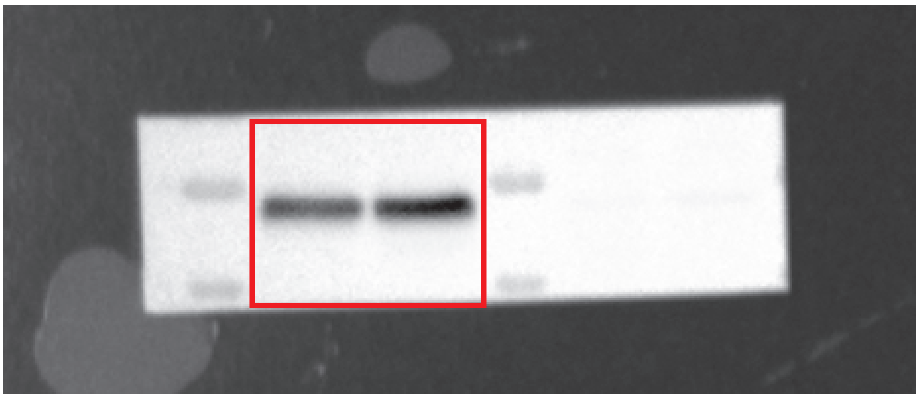

Figure 1G-Nanog

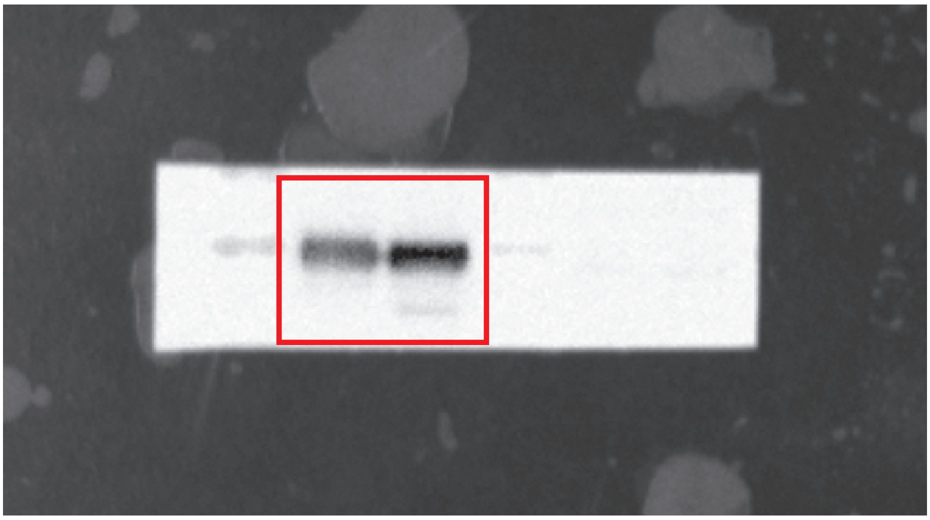

Figure 1G-GAPDH

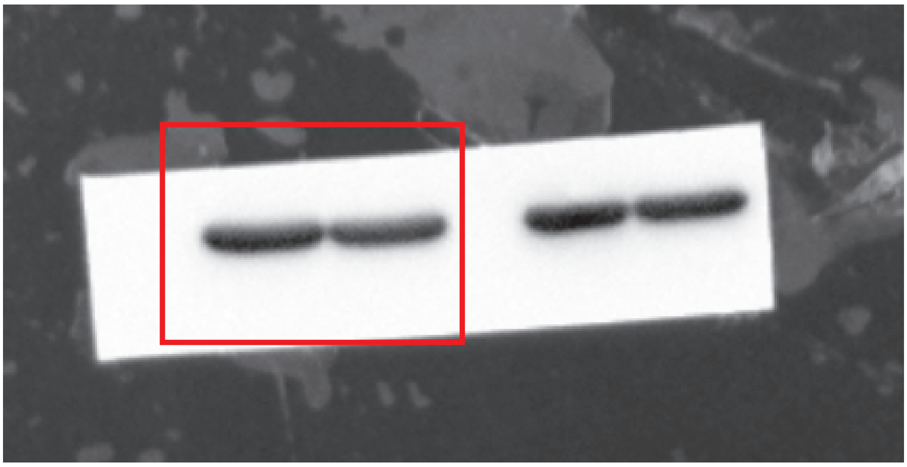

Figure 4

Figure 4E-MACC1

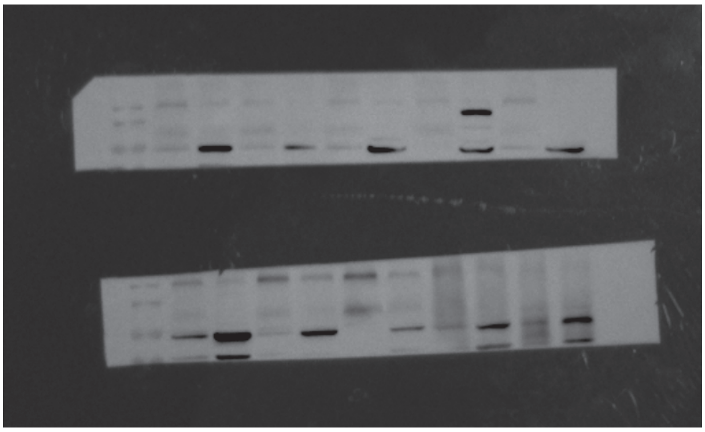

Figure 4E-KLF4-left

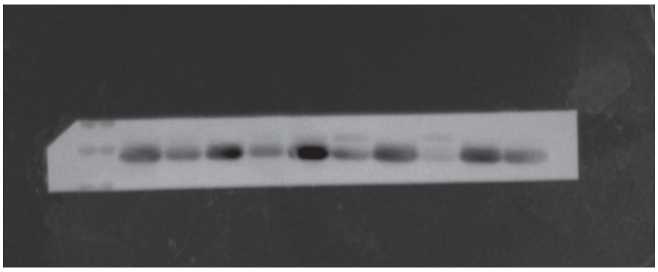

Figure 4E-KLF4-right

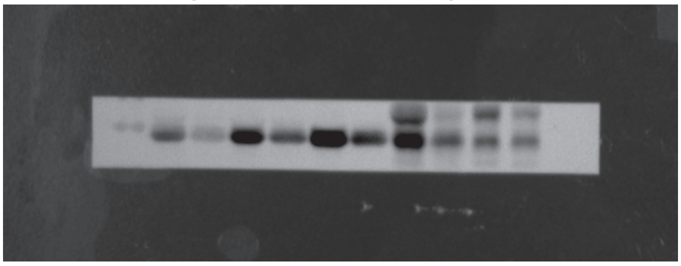

Figure 4E-GAPDH-left

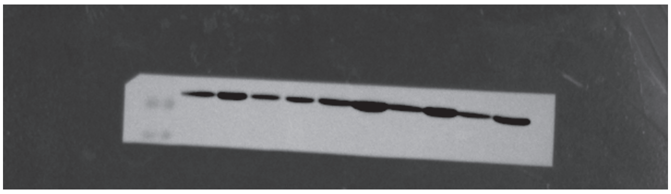

Figure 4E-GAPDH-right

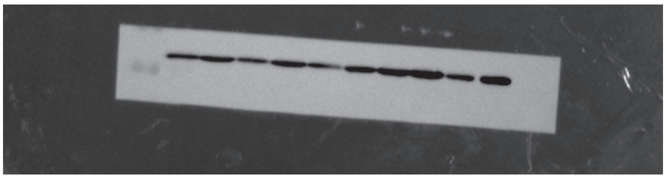

Figure 5

Figure 5A-MACC1

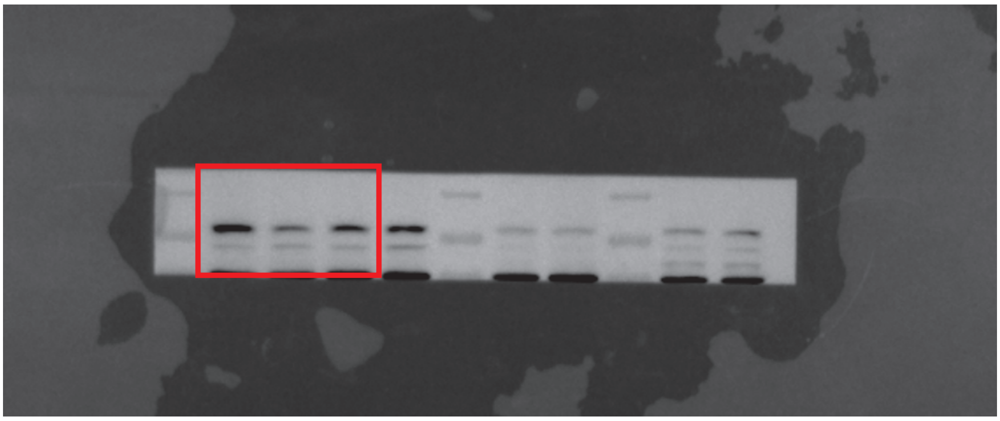

Figure 5A-KLF4

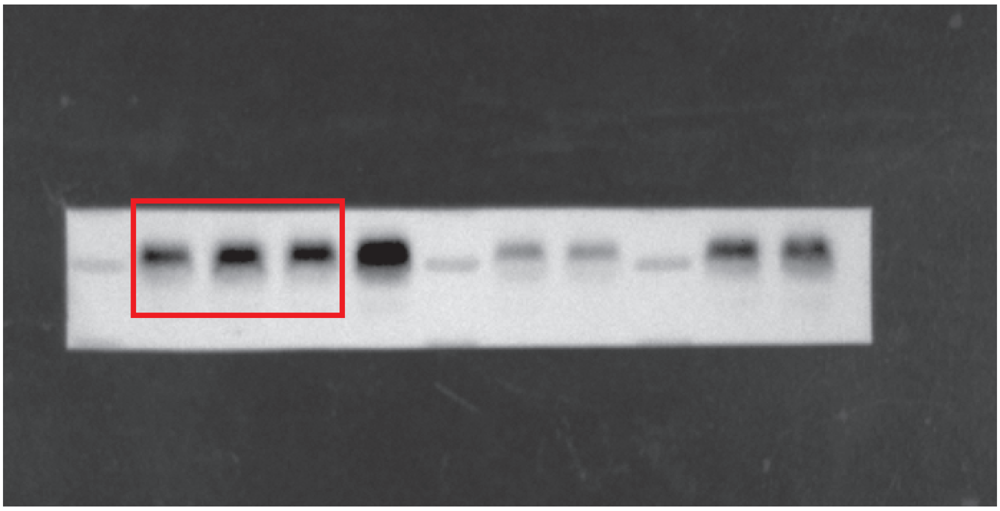

Figure 5A-GAPDH

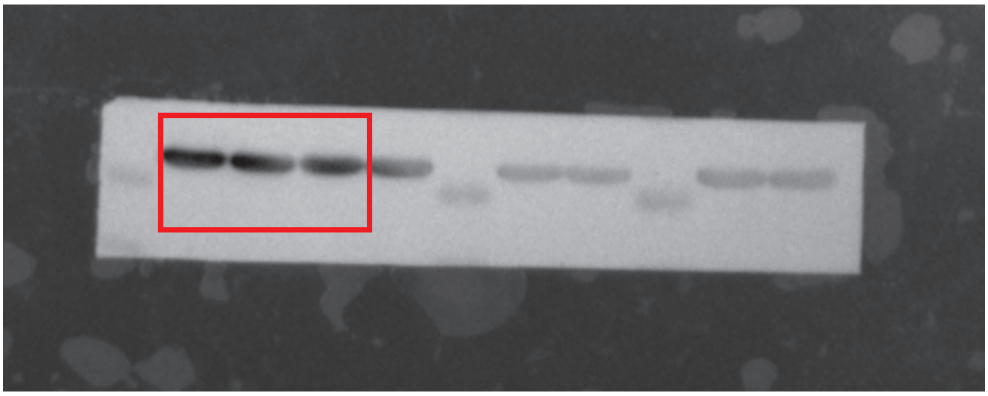

Figure 5E-MACC1

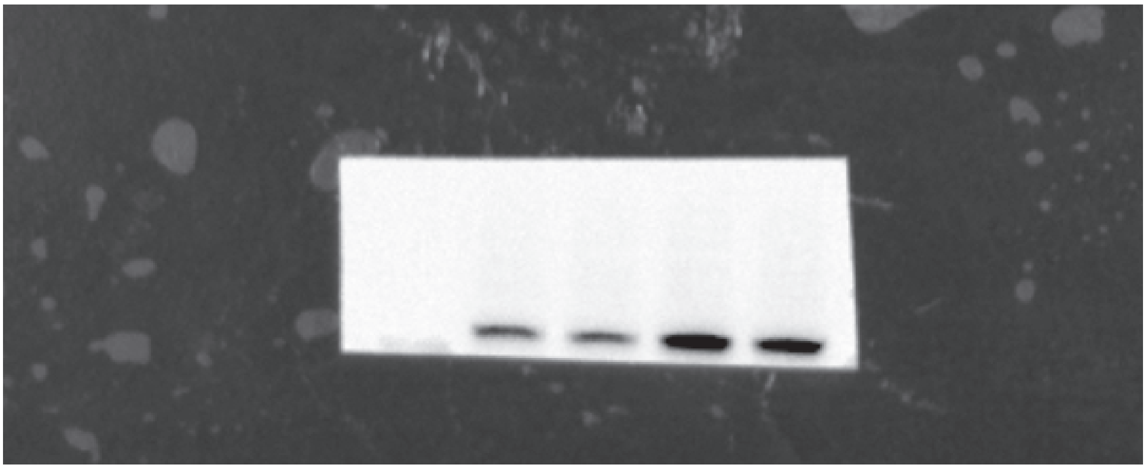

Figure 5E-KLF4

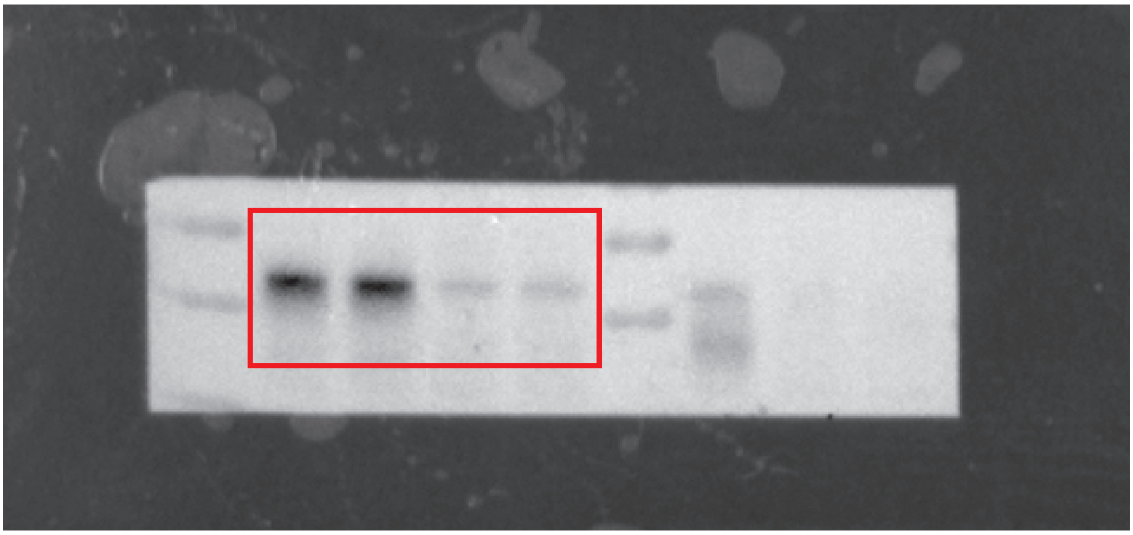

Figure 5E-GAPDH

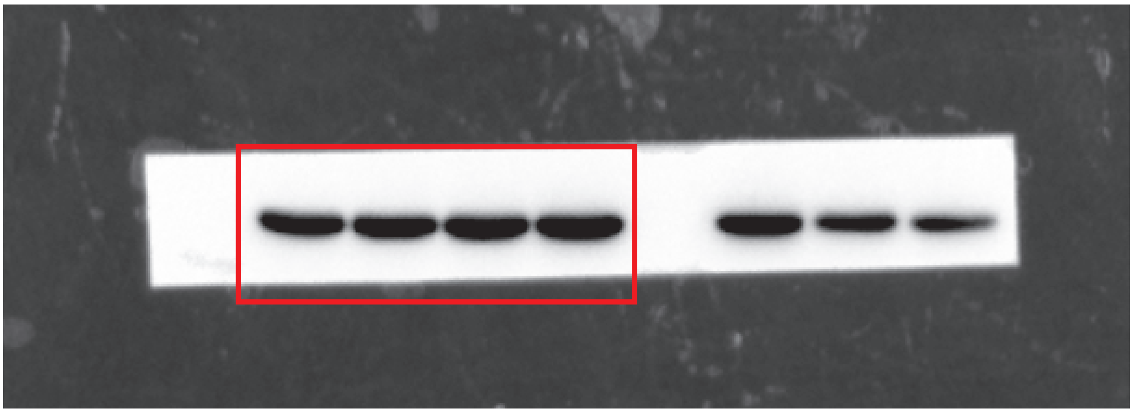

Figure 6

Figure 6J-MACC1

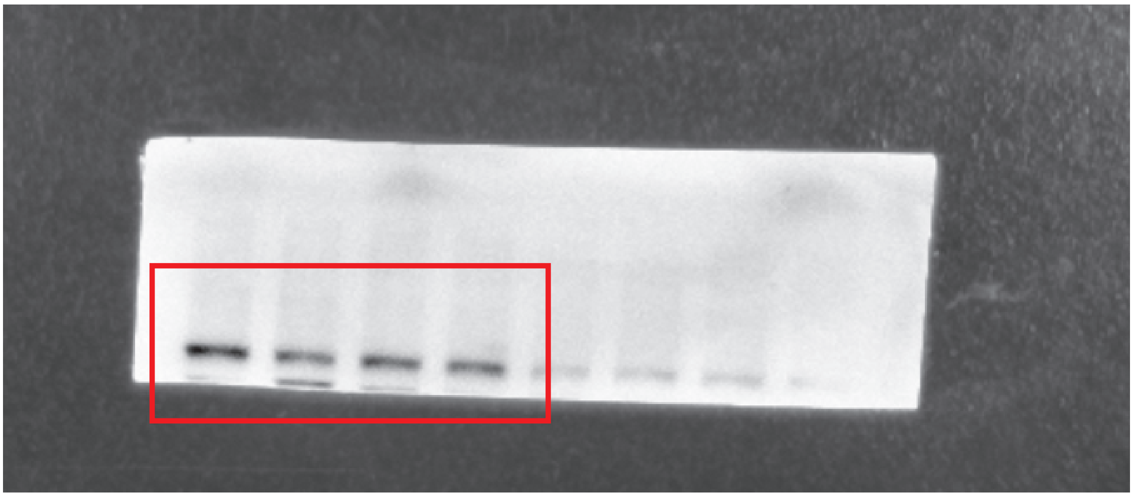

Figure 6J-KLF4

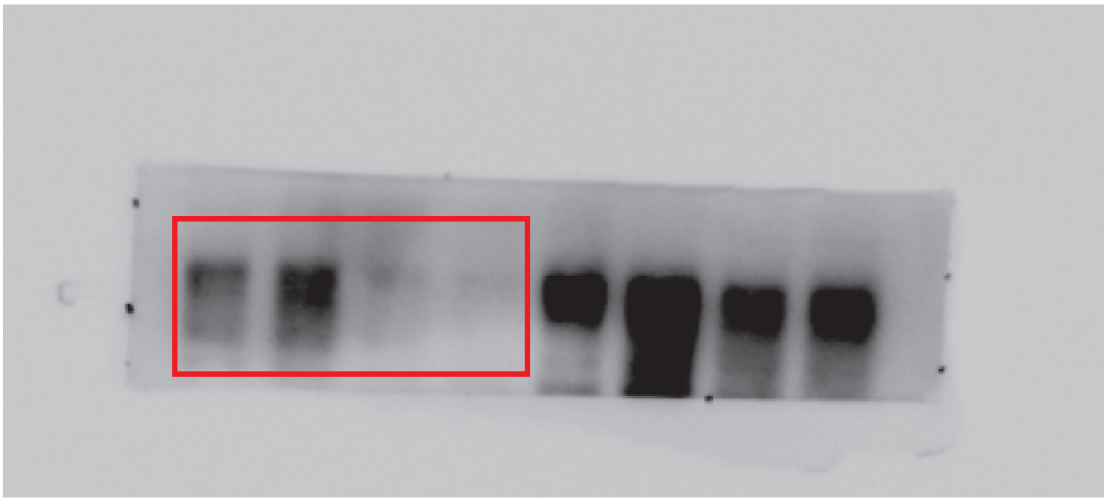

Figure 6J-GAPDH

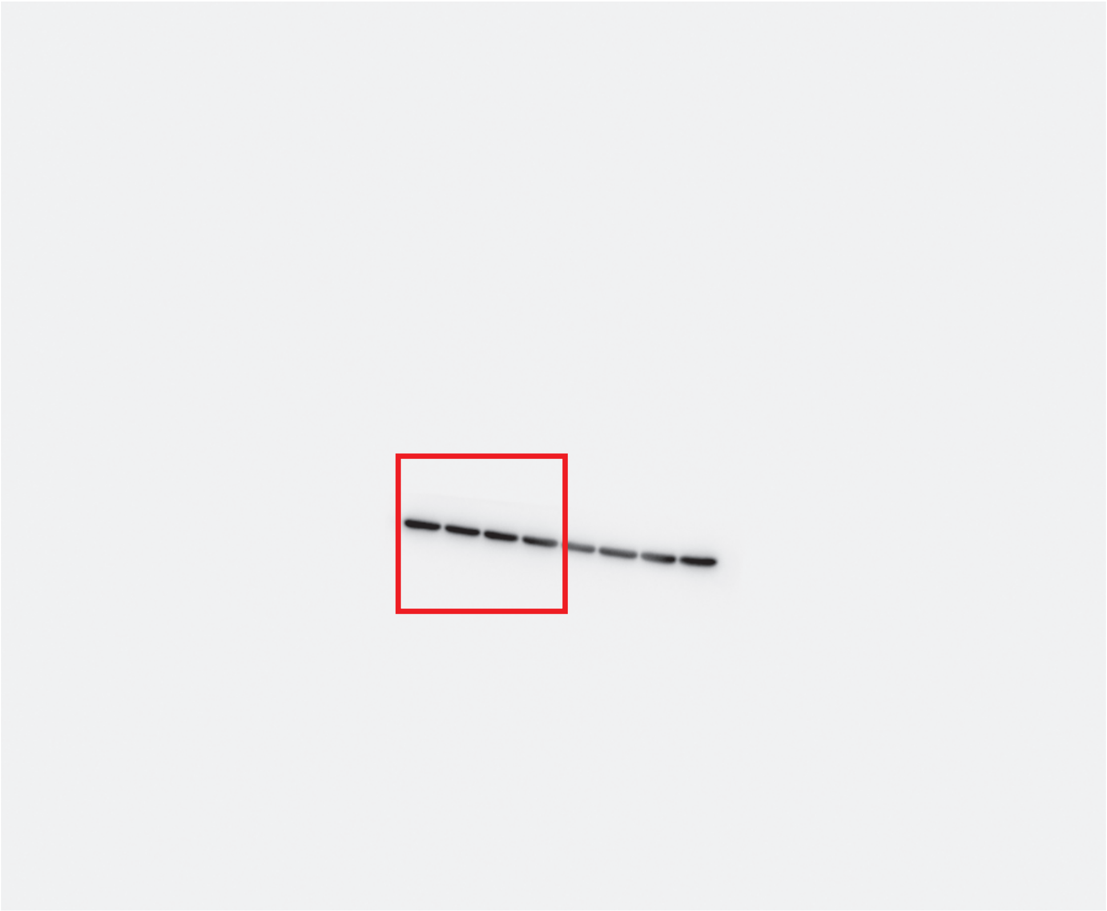

Figure 6L-MACC1

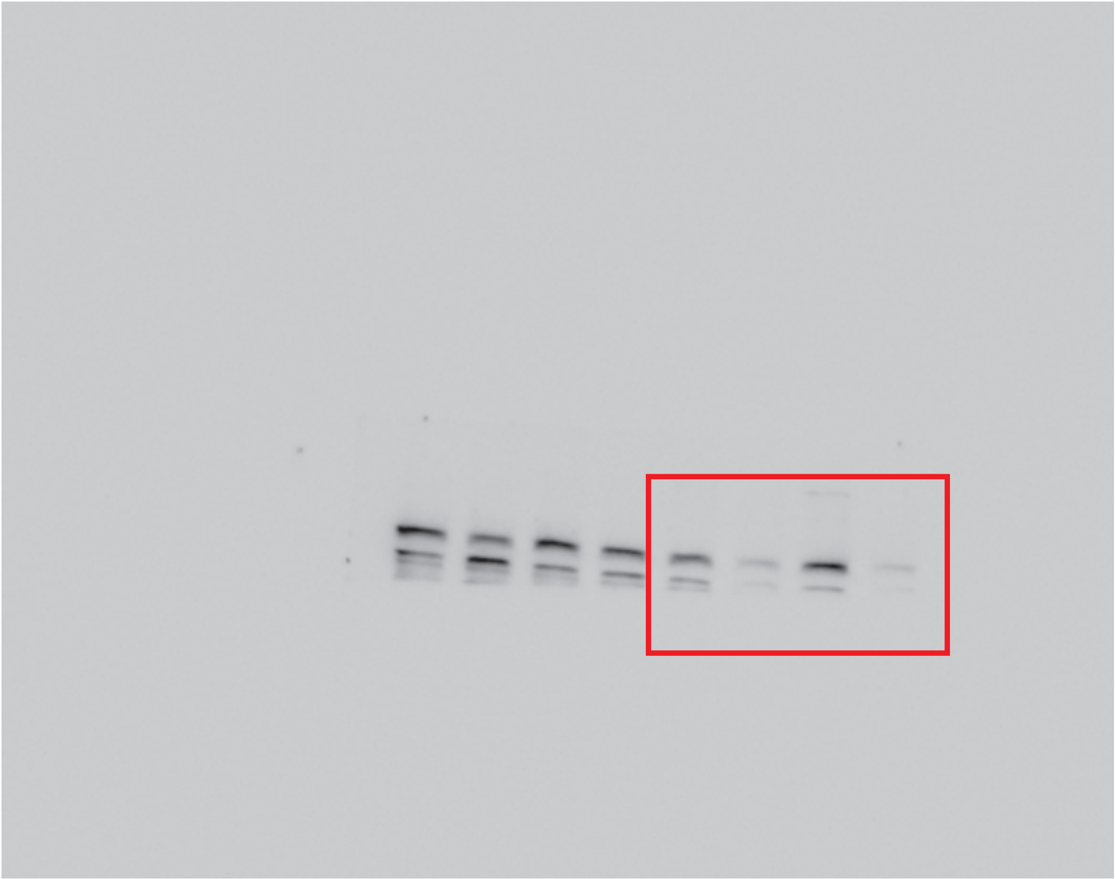

Figure 6L-KLF4

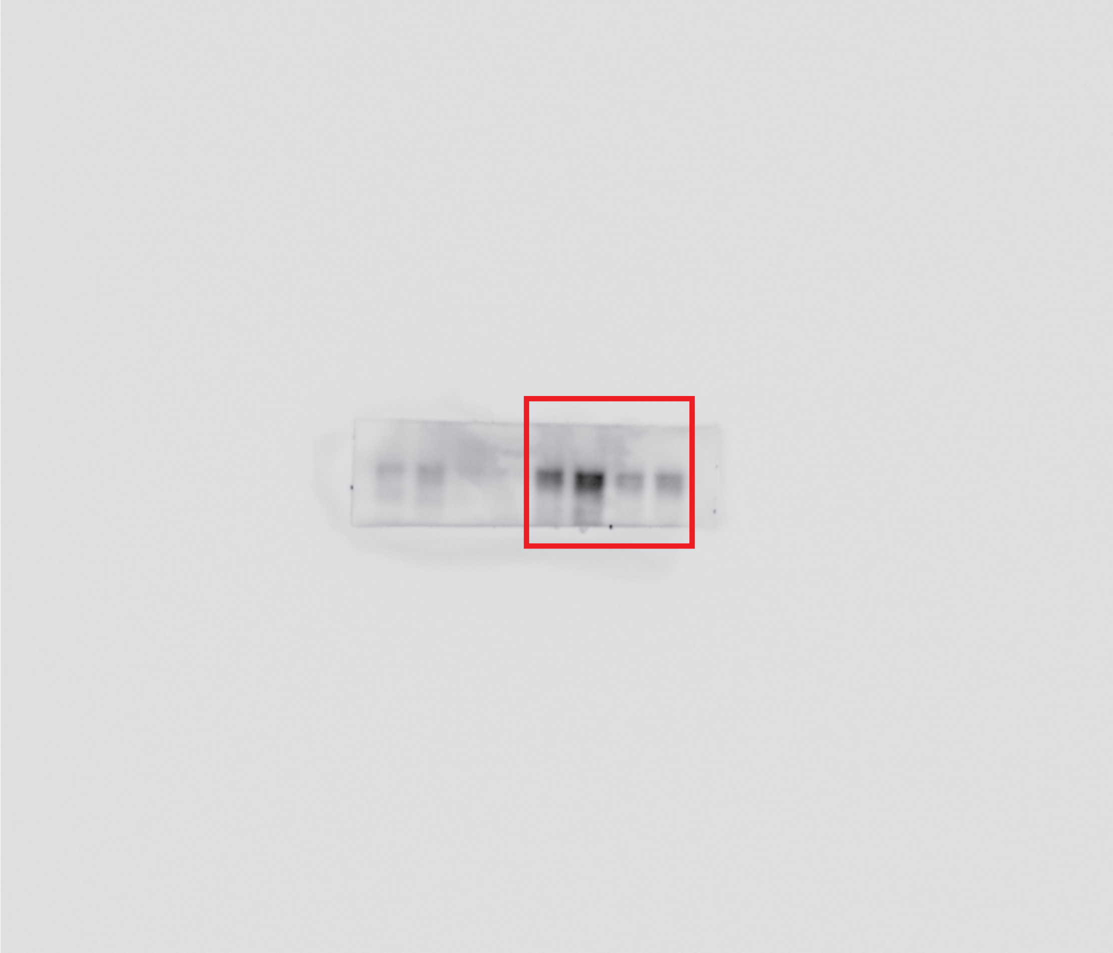

Figure 6L-GAPDH

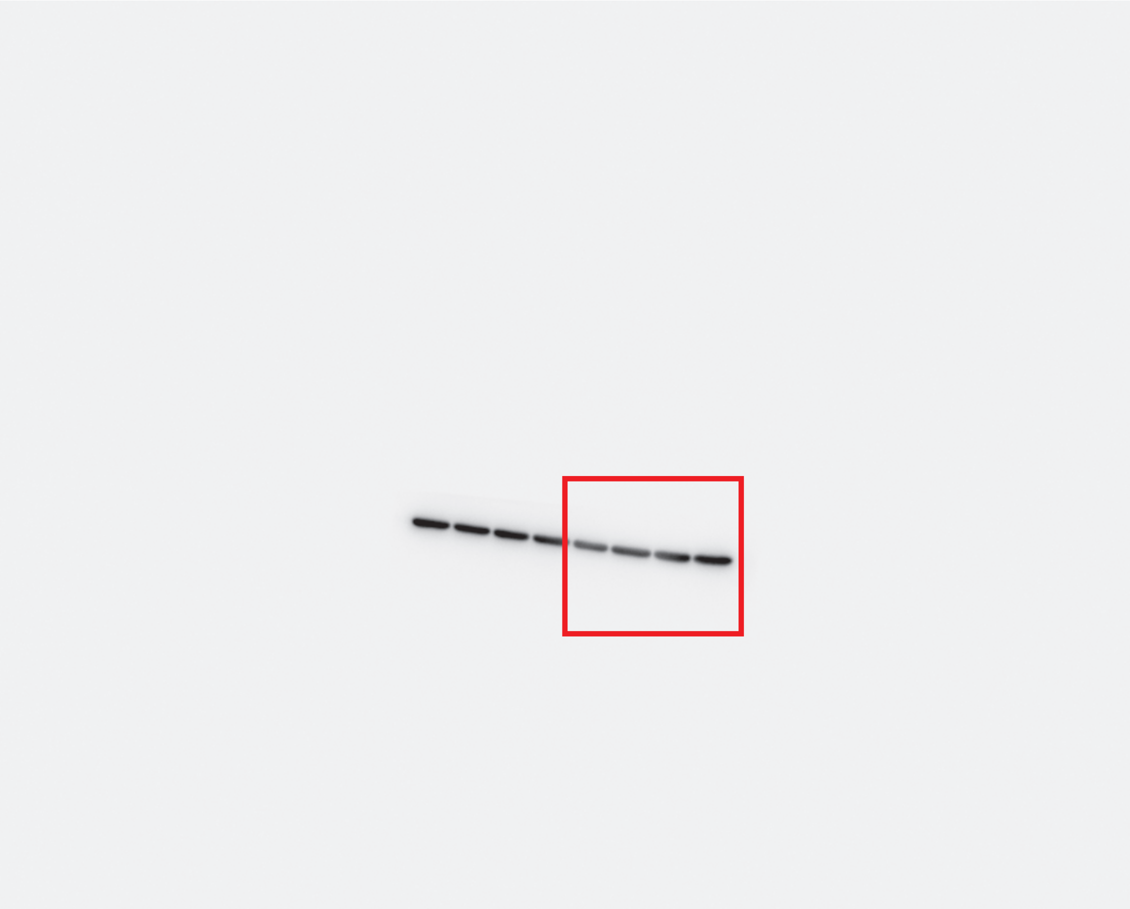

Supplementary Figure 1

Supplementary Figure 1A-MACC1

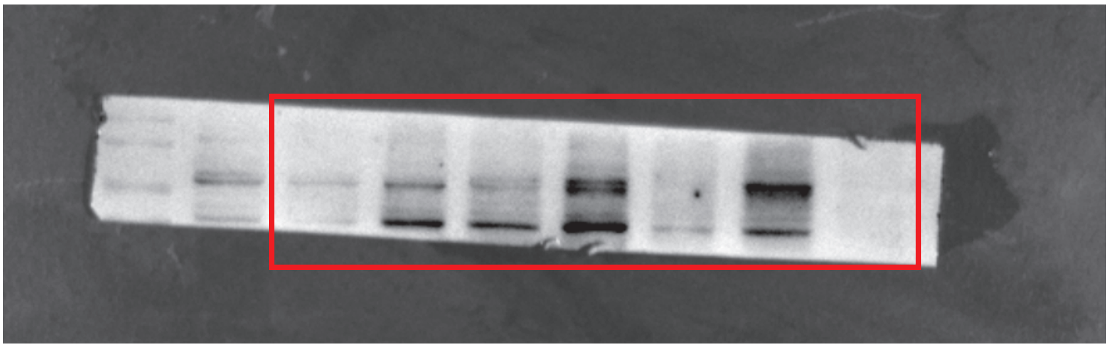

Supplementary Figure 1A-GAPDH

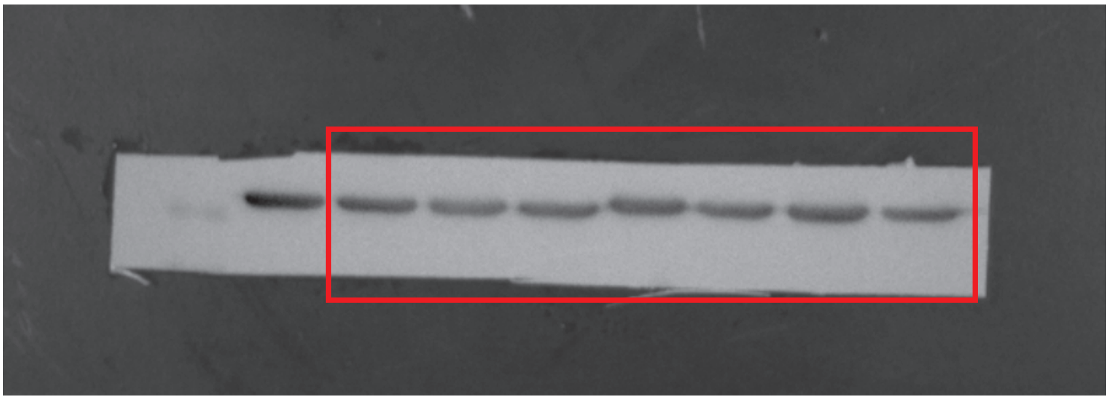

Supplementary Figure 1B-HA

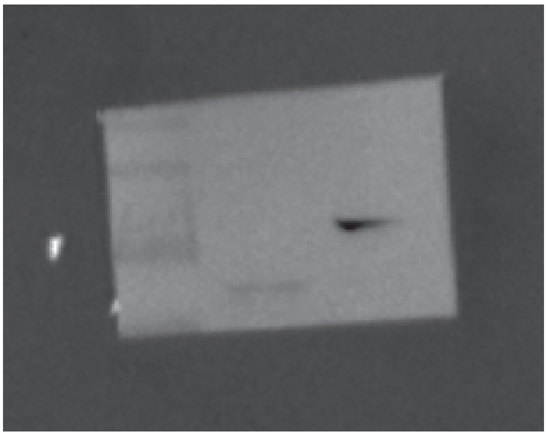

Supplementary Figure 1B-MACC1

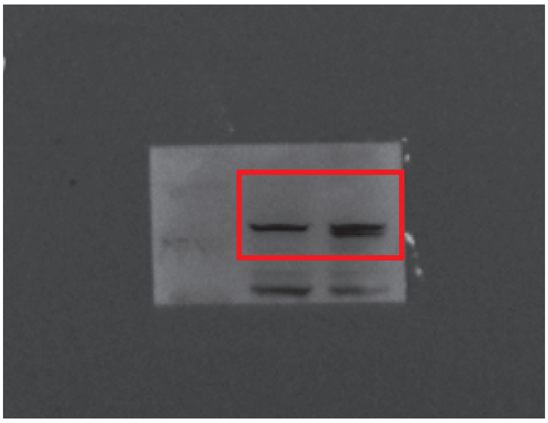

Supplementary Figure 1B-GAPDH

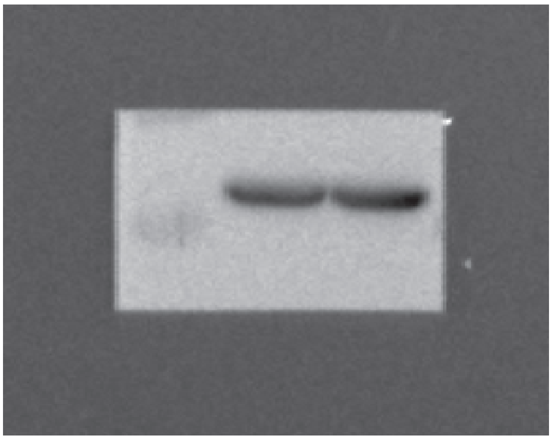

Supplementary Figure 1C-MACC1

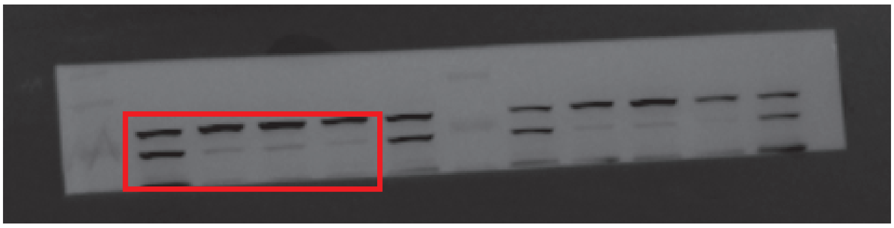

Supplementary Figure 1C-GAPDH

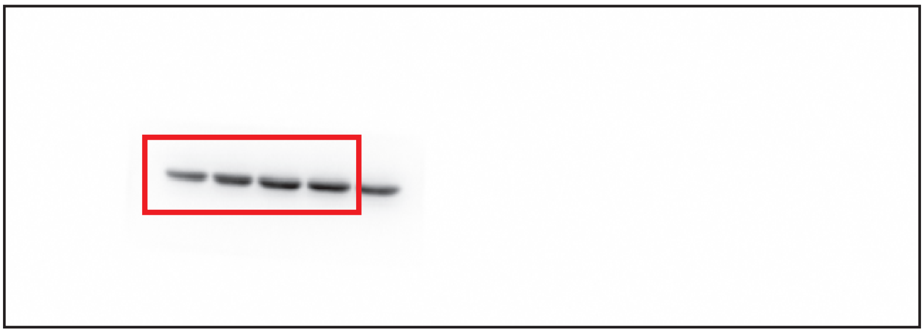

Supplementary Figure 3

Supplementary Figure 3A-MACC1

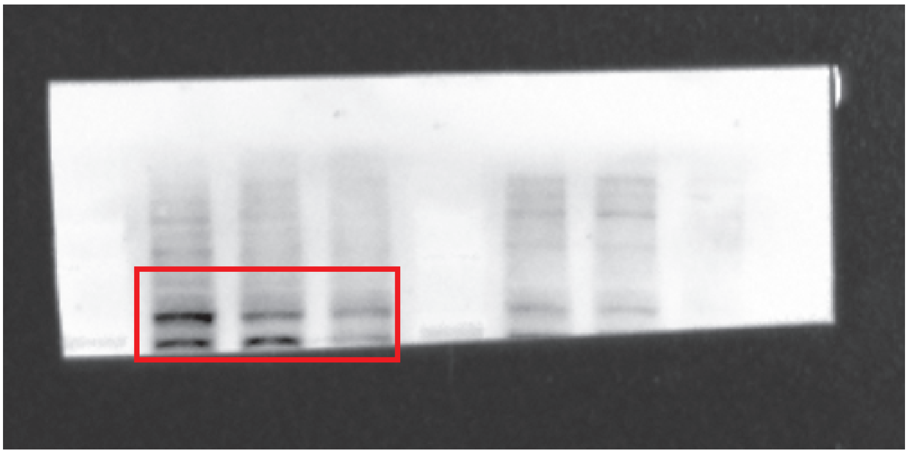

Supplementary Figure 3A-KLF4

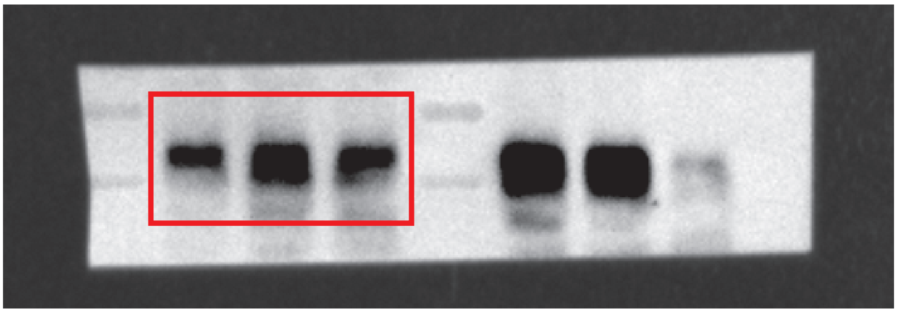

Supplementary Figure 3A-GAPDH

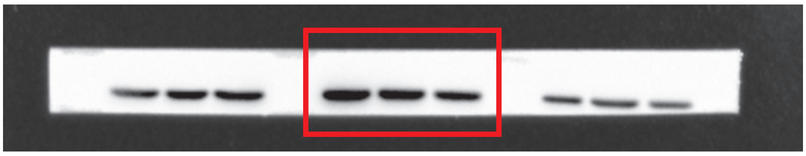

Supplementary Figure 3E-MACC1

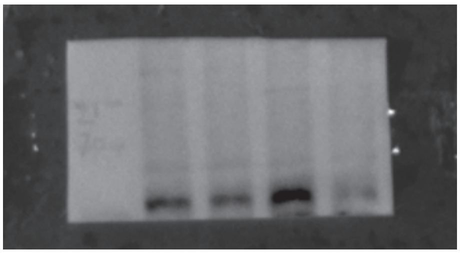

Supplementary Figure 3E-KLF4

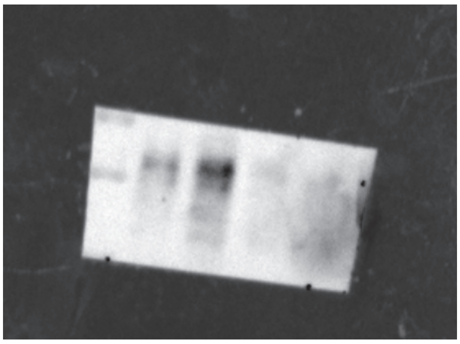

Supplementary Figure 3E-GAPDH

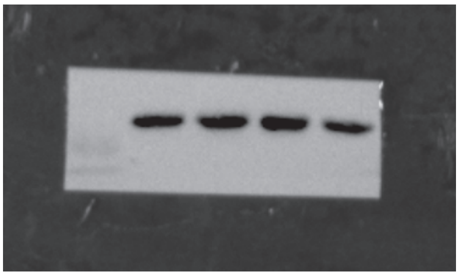

Supplement: Supplementary file 3 — Full and uncropped western blots [file 41420_2024_2256_MOESM3_ESM.pdf]
